# Melatonin promotes seed germination under salinity and enhances the biosynthesis of steviol glycosides in *Stevia rebaudiana* Bertoni leaves

**DOI:** 10.1371/journal.pone.0230755

**Published:** 2020-03-27

**Authors:** Magdalena Simlat, Agnieszka Szewczyk, Agata Ptak

**Affiliations:** 1 Department of Plant Breeding, Physiology and Seed Science, University of Agriculture in Krakow, Krakow, Poland; 2 Department of Pharmaceutical Botany, Faculty of Pharmacy, Jagiellonian University Medical College, Krakow, Poland; Hainan University, CHINA

## Abstract

Melatonin (MEL) can act as a plant growth regulator and biostimulator in stressful situations. Using MEL in seed pretreatment also affects the future growth of plants. Therefore, this research investigated the effects of MEL on seed germination and seedling growth under NaCl in in vitro conditions. The additional effects of MEL on the accumulation of steviol glycosides (SGs) and on the expression of appropriate genes were also studied. Five μM of MEL was the best concentration for seed germination, while 20 μM exerted a positive impact on the biomass of stevia plantlets. NaCl significantly decreased seed germination, but MEL alleviated this effect when seeds were germinated in 50 mM of NaCl. Under salinity, the values of almost all morphological traits decreased as MEL concentration increased. The highest amounts of stevioside and rebaudioside A (Reb A) were observed as a result of treating seeds with 5 and 20 μM of MEL, respectively. When adding NaCl, positive impacts of MEL on the accumulation of both SGs were also observed. Expression analyses of the genes involved in SGs biosynthesis was explored in seeds and leaves, and the transcripts of key enzymes occurred in both the tissues. However, quantitative polymerase chain reaction (qPCR) analysis showed that all tested genes were upregulated in younger leaves, contrary to older ones. Also in younger, rather than older, leaves SG gene expression varied according to MEL concentration. This study, therefore, presents the promising potential of MEL for improving stevia seed germination under salinity conditions and for enhancing the production of SGs in stevia plants.

## Introduction

Melatonin (MEL) (N-acetyl-5-methoxytryptamine) has been detected in many plant species, including both monocotyledonous and dicotyledonous families [1–4]. MEL is called a multiregulatory molecule [5] due to its broad spectrum of activity throughout the life of a plant [6–8]. Exogenous MEL, used for seed germination, also affects the future growth of plants. In canary grass, wheat, barley, and oats, MEL stimulates stem elongation [6], and, in lupin, it stimulates cotyledon elongation [9]. The effect of MEL on leaf and root development, as well as on plant reproductivity, has also been described in previous research [8, 10–13], and the positive impact of MEL on germination and growth has been especially reported under stress conditions [14, 15]. Salinity is currently a critical agricultural problem, which leads to low plant production. Salinity stress, because of the accumulation of free radicals in plants, can negatively affect seed germination, resulting in reduced plant growth [16]. Various approaches, including applying MEL, have been tested to improve plant response to salt stress [17–19]. Recently published studies have shown that MEL also affects the levels of valuable secondary metabolites in in vitro cultures of *Rosmarinus officinalis* [20] and *Leucojum aestivum* [21].

**Fig 1:**
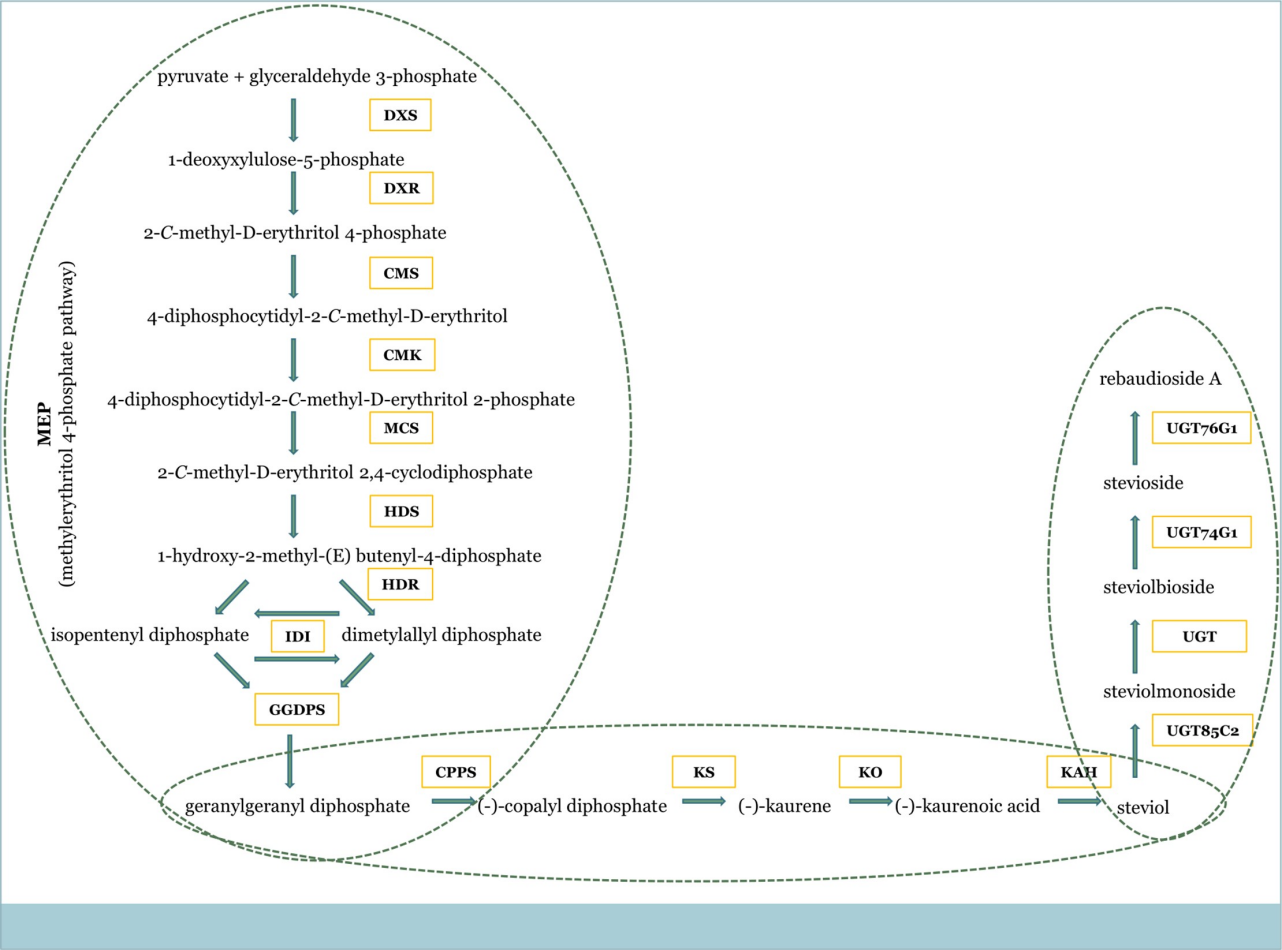
The biosynthesis pathway of steviol glycosides in *Stevia rebaudiana* Bertoni plant. DXS 1-deoxyxylulose-5-phosphate synthase; DXR 1-deoxyxylulose-5-phosphate reductoisomerase; MCT 4-diphosphocytidyl-2*C*-methyl-D-erythritol synthase; CMK 4-diphosphocytidyl-2*C*-methyl-D-erythritol kinase; MDS 4-diphosphocytidyl-2*C*-methyl-D-erythritol 2,4-cyclodiphosphate synthase; HDS– 1-hydroxy-2-methyl-2(E)-butenyl 4-diphosphate synthase; HDR 1-hydroxy-2-methyl-2(E)-butenyl 4-diphosphate reductase; IDI isopentenyl diphosphate isomerase; GGDPS geranylgeranyl diphosphate synthase; CPPS1 copalyl diphosphate synthase; KS kaurene synthase; KO kaurene oxidase; KAH kaurenoic acid hydroxylase; UGT85C2 UDP-glycosyltransferase-85C2; UGT74G1 UDP-glycosyltransferase-74G1; UGT76G1 UDP-glycosyltransferase-76G1.

Stevia (*Stevia rebaudiana* Bertoni) is a member of the Asteraceae family, which accumulates steviol glycosides (SGs), a kind of tetracyclic diterpene derived through the 2-*C*-methyl-D-erythritol 4-phosphate (MEP) pathway (Fig 1). Steviol glycosides are approximately 200 times sweeter than common sugar but much less calorific [22]. As high-potency sweeteners, SGs are recommended for patients with diabetes and phenylketonuria [23]. Among the 30 different SGs synthesized in stevia leaves, stevioside and rebaudioside A (Reb A) dominate. Although stevia is naturally propagated by seeds, due to poor germination, seed propagation is not a widespread method for commercial stevia production. However, some reports on the possibility of improving stevia seed germination have already been published [24–27].

The positive effects of MEL on seed germination and plant growth, especially under stress conditions, has been proven for several species. Therefore, this study hypothesized that MEL would also enhance the germination of stevia seeds under NaCl conditions and would affect the further growth of the obtained seedlings, as well as the biosynthesis of SGs in stevia leaves.

## Materials and methods

### Seed germination

For these experiments, the seeds (POLAN Breeding and Seed Company, Poland) were first surface-sterilized according to Simlat et al. [26]. After sterilization, the seeds were dried on filter paper until they returned to their initial water content (12%). They were then used to conduct three germination experiments and gene expression analyses.

In the first experiment, dried seeds were incubated in aqueous solutions of MEL (M5250; Sigma-Aldrich): 5 μM (5MEL), 20 μM (20MEL), 100 μM (100MEL), and 500 μM (500MEL). For the control (0MEL), distilled H_2_O (dH_2_O) was used. Incubation lasted 24 h in the dark at 25°C, during which the seeds were mixed (130 rpm). After incubation, dried seeds (as described above) were placed in Petri dishes containing agar gel (AG) (dH_2_O solidified with 0.7% Difco Bacto Agar).

In the second experiment, dried seeds were placed in Petri dishes containing AG supplemented with different concentrations of NaCl (S3014, Sigma-Aldrich): 50 mM, 100 mM, 150 mM, and 200 mM. Petri dishes containing AG without NaCl (0 mM NaCl) were used as the control.

In the third experiment, dried seeds were first incubated in aqueous solutions of MEL (see description of first experiment) and were then placed in Petri dishes containing AG supplemented with selected NaCl concentrations: 50 mM and 150 mM. Germination in AG without NaCl (0 mM NaCl) was used as a control.

Agar gel and AG with NaCl were sterilized by autoclaving, while MEL solutions were drawn from a 10 mM stock solution, filter-sterilized (Millex–GP, 0.22 μm, Millipore). In all three experiments, the germination conditions were the same and were in accordance with Simlat et al. [27]. The experiments were performed in four replications, each consisting of 25 seeds. Every day, during 21 days of germination, the number of germinated seeds was recorded. After seven and 21 days, germination energy (GE; %) and germination capacity (GC; %) were determined, respectively.

### Seedling growth

Well-developed seedlings obtained in the first and third germination experiments were transferred to a Murashige and Skoog (MS) medium [28] for further growth. After four weeks, the morphologies of five randomly chosen plantlets were assessed. Growth conditions and morphology assessments were performed according to Simlat et al. [27]. The remaining plantlets were transplanted into pots (15 x 15 cm) containing soil, peat, and sand in a 3:1:1 ratio for further growth (25°C, fluorescent light with intensity expressed as PPFD of 320 *μ*mol m^-2^s^-1^ for 16 h/day at 70±5% relative humidity; Adaptis-A1000AR, Conviron). After six months of growth, younger and older leaves were collected as separate samples and were stored at -80°C until biochemical and molecular analyses were conducted. Younger leaves were picked from the highest three nodes of a plant, whilst older leaves were collected from nodes fourth and lower.

### High-Performance Liquid Chromatography (HPLC) analysis of steviol glycosides

For analysis, 250 mg of dry biomass was extracted with 2 ml of methanol in an ultrasonic bath for 1 h at 30°C. Obtained extracts were filtered through a PTFE, 0.22 μm Millipore filter. Reversed-phase (RP)-HPLC analysis was conducted as described by Bayraktar et al. [29], with our modification, on a Merck-Hitachi liquid chromatograph (LaChrom Elite) equipped with a DAD detector L-2455 and Purospher RP-18e column. Analysis was carried out at 40°C with a mobile phase consisting of A—water and B—acetonitrile. The gradient was as follows: 10–20% B for 0–2 min; 20–35% B for 2–6 min; 35–40% B for 6–9 min; 40–50% B for 9–10 min; 50–80% B for 10–15 min; 80% B for 15–20 min; 80–10% B for 20–40 min; 10% B for 40–45 min at a flow rate 1 ml min^-1^. Detection was performed at λ = 210 nm, and the UV spectra of all samples were scanned between 190 nm and 360 nm. Quantification was defined by measuring the peak area with reference to the standard curve derived from five concentrations (from 0.0625 mg ml^-1^ to 1 mg ml^-1^) of authentic reference compounds: stevioside (50956; Sigma-Aldrich) and Reb A (38462; Sigma-Aldrich).

### RNA isolation and expression analyses of steviol glycoside biosynthesis pathway genes

Total RNA was isolated from the 20 seeds or 100 mg of the leaf tissue powder using the Spectrum Plant Total RNA Kit (STRN250; Sigma-Aldrich) according to the manufacturer’s instruction. RNA was eluted by 30 μl of Elution Buffer and stored at -80°C until use. The quality and quantity of the RNA were monitored using a spectrophotometer (NanoDrop 2000c, Thermo Scientific) and via standard agarose electrophoresis. To synthesize the first cDNA strand, an equal amount of 1 μg of RNA was used. Prior to reverse transcription (RT), RNA was incubated with DNaze I (EN0521; Thermo Scientific) (2 U DNaze I/1 μg RNA) for 30 min at 37°C. Reverse transcription was carried out using the Maxima^TM^ First Strand cDNA Synthesis Kit (K1641; Thermo Scientific) at 42°C in 20 μl reactions, according to the manufacturer’s instructions. Polymerase chain reaction (PCR) was performed using 1 μl of 2.5-fold diluted cDNA in 20 μl of reaction mixture, which contained 1 x *Taq* buffer, 2.5 mM MgCl_2_, 0.25 mM of each dNTP, 0.25 μM each of forward and reverse primer, and 1 U *Taq* DNA polymerase (EP0405, Thermo Scientific). Primers sequences [30, 31], given in the Table 1, were synthesized in Genomed (Poland). The *actin* gene was used as reference. The PCR conditions (Master Gradient Eppendorf) were as follows: 94°C for 5 min, 35 cycles at 92°C for 45 s, 57°C for 45 s, 72°C for 2 min, and 72°C for 10 min. The products of PCR were separated by electrophoresis in 1.5% agarose gel and visualized by ethidium bromide (E7637, Sigma-Aldrich). These RT-PCR experiments were repeated three times.

**Table 1:** Primers used in RT-PCR and qPCR experiments.

Gene	Accession number (NCBI)	Primer sequence 5’→3’	Amplicon length(bp)	Reference
SrACT* (reference)	AF548026	F: actatgaattgcccgatggt	267	Designed be the author of that publication
		R: tgatcttcatgctgctaggg		
*SrDXS*	AJ429232	F: gatctacaaaagttaccggttc	476	[30]
		R: tcctctacggtaagtaagacttc		
*SrDXR*	AJ429233	F: ttgagctatctatctccaacac	354	
		R: tatctgttcagcaagaagagtc		
*SrMCT*	DQ269452	F: agacaagattctgtttttagtg	394	
		R: gagttgtaaccttgatgttagt		
*SrCMK*	DQ269453	F: aatctatatcgcaagaagactg	536	
		R: cttccagacataaaaacagaat		
*SrMDS*	DQ631427	F: gagcctggataccctctcatc	295	
		R: cctctttatgcgggcttaact		
*SrHDS*	DQ768749	F: aaaaggttgattgatgtaagtat	426	
		R: taataagataccatctccaagtc		
*SrHDR*	DQ269451	F: aaacaatttgatgtcattgataa	465	
		R: ggttctttctactagttttccaa		
SrIDI*	DQ989585	F: tatgagttactccttcagcaac	269	
		R: aggtagtcaagttcatgttctc		
SrGGDPS*	DQ432013	F: agttcatgacgaccttccatgca	291	
		R: atatgaatatactccaagtcgtc		
SrCPPS1*	AF034545	F: cgactcgagacaagatattact	382	
		R: ctataaaggctgttatgtcctc		
*SrKS1-1*	AF097310	F: gagagaagctatatggacaagag	493	
		R: gatgtccttcacagtatcaaga		
*SrKO1*	AY364317	F: gttgaaggagaagaaaccttac	464	
		R: caacatataagctctccacatc		
SrUGT85C2*	AY345978	F: tcgatgagttggagcctagtatt	153	[31]
		R: ctaaactgtatccatggagactc		
SrUGT74G1*	AY345982	F: tgcatgaactggttagacgataag	274	
		R: gcatcctactgattcgtgtgcta		
SrUGT76G1*	AY345974	F: gcagcttactagaccacgatc	107	
		R: ctcatccacttcactagtactac		

*Sr*–*Stevia rebaudiana*

NCBI—The National Center for Biotechnology Information (https://www.ncbi.nlm.nih.gov)

*—chosen for qPCR analysis; F–forward; R—reverse

ACT–β-actin; DXS 1-deoxyxylulose-5-phosphate synthase; DXR 1-deoxyxylulose-5-phosphate reductoisomerase; MCT 4-diphosphocytidyl-2*C*-methyl-D-erythritol synthase; CMK 4-diphosphocytidyl-2*C*-methyl-D-erythritol kinase; MDS 4-diphosphocytidyl-2*C*-methyl-D-erythritol 2,4-cyclodiphosphate synthase; HDS– 1-hydroxy-2-methyl-2(E)-butenyl 4-diphosphate synthase; HDR 1-hydroxy-2-methyl-2(E)-butenyl 4-diphosphate reductase; IDI isopentenyl diphosphate isomerase; GGDPS geranylgeranyl diphosphate synthase; CPPS1 copalyl diphosphate synthase; KS1-1 kaurene synthase; KO1 kaurene oxidase; UGT85C2 UDP-glycosyltransferase-85C2; UGT74G1 UDP-glycosyltransferase-74G1; UGT76G1 UDP-glycosyltransferase-76G1.

Quantitative PCR (qPCR) was performed using the 7500 Fast Real Time PCR System (Applied Biosystems). For amplification, the reaction mixture included 2.5 μl of 20-fold diluted cDNA, 2X Fast SYBR Green Master Mix (4385612, Applied Biosystems), and 0.5 μM each of forward and reverse primer (Table 1). The following thermal profile was used for all reactions: 95°C for 20 s, 40 cycles of 95°C for 3 s per cycle, and 60°C for 1 min per cycle. Melting curve analysis was conducted to evaluate the presence of non-specific PCR products and primer dimmers (S1 Fig). The experiment was performed in three technical and three biological replications. The obtained data were analyzed using the 7500 Software v2.0 (Applied Biosystems) based on the 2^-ΔΔCt^ method [32]. The *actin* gene was used as an internal reference, and the relative transcript level was normalized for each sample to that of control older leaves (0MEL).

### Statistical analysis

The data were reported as means ± standard deviation (SD). Two-factorial analysis of variance (ANOVA) was performed using STATISTICA (data analysis software system, version 10.0; www.statsoft.com) with MEL and NaCl concentrations as the factors. To compare means, Duncan’s multiple range test was used at a significance level of P < 0.05 implemented in the statistical package. Correlation between the content of steviol glycosides and expression of their biosynthesis genes was analyzed using Microsoft Excel.

## Results

### The effect of MEL on seed germination (the first germination experiment)

**Fig 2:**
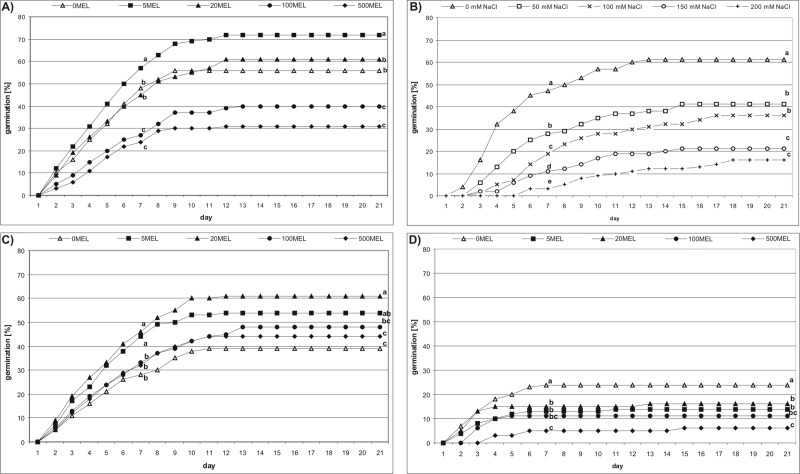
The effect of MEL and NaCl on stevia seed germination. Seeds were soaked in MEL solutions (0MEL, 5MEL, 20MEL, 100MEL and 500MEL) and germinated on AG (A); seeds without MEL treatment were germinated on AG with NaCl (0, 50, 100, 150 and 200 mM) (B); seeds soaked in MEL solutions were germinated on AG with 50 mM NaCl (C) or on AG with 150 mM NaCl (D). The results are means of four replicates (n = 4), each replication consisting of 25 seeds. Different letters indicate a significant difference in a percentage of germinated seeds at 7^th^ (germination energy; GE) and 21^st^ (germination capacity; GP) day of incubation at P < 0.05 according to ANOVA and Duncan’s test.

Soaking seeds with 5MEL had the most favorable effect on germination (Fig 2A). This effect was observed from the second day of germination. Consequently, both germination energy (GE) and germination capacity (GC) had the highest values at this concentration: 57% and 72%, respectively. Considering the remaining MEL solutions, seed germination significantly decreased (P < 0.05) as MEL concentration increased. For 500MEL, GC amounted to 31%, but, for the control (0MEL), it was 56%. Thus, the effect of MEL concentration on seed germination was significant (P < 0.001) (Table 2).

**Table 2:** The results of two-factorial analysis of variance (ANOVA). Differences in germination energy (GE), germination capacity (GP) were analyzed with MEL (0, 5, 20, 100 and 500 μM) and NaCl (0, 50, 150 mM) as factors. Seedlings weight, stem and root length, leaves and roots number, stevioside and rabaudioside A (Reb A) content were analyzed with MEL (0, 5, 20, 100 and 500 μM) and NaCl (0, 50 mM) as factors.

	Source	df	MS	F-value
GE (%)	MEL concentration (M)	4	730.930	33.291***
	NaCl concentarion (S)	2	4139.470	188,538***
	M x S	8	262.130	11.939***
	Error	45	21.960	
GC (%)	MEL concentration (M)	4	860.400	26.090***
	NaCl concentarion (S)	2	8872.270	269.038***
	M x S	8	346.600	10.510***
	Error	45	32.980	
Fresh weight (g)	M	4	0.198	29.5654***
	S_1_	1	0.013	2.0077^ns^
	M x S_1_	4	0.200	29.8643***
	Error	40	0.007	
Shoot fresh weight (g)	M	4	0.079	45.6312***
	S_1_	1	0.025	14.2022***
	M x S_1_	4	0.113	65.0072***
	Error	40	0.002	
Stem length (cm)	M	4	3.143	8.2139***
	S_1_	1	14.125	36.9090***
	M x S_1_	4	3.582	9.3612***
	Error	40	0.383	
Leaves/plantlets number	M	4	8.280	5.595**
	S_1_	1	54.080	36.541***
	M x S_1_	4	8.280	5.595**
	Error	40	1.480	
Roots number	M	4	28.819	5.9313***
	S_1_	1	4.061	8.8359^ns^
	M x S_1_	4	45.899	9.4466***
	Error	40	4.859	
Root length (cm)	M	4	33.634	48.8439***
	S_1_	1	0.871	1.2652^ns^
	M x S_1_	4	4.178	6.0679***
	Error	40	0.689	
Stevioside–older leaves	M	4	11.690	50.644***
	S_1_	1	2.642	11.447**
	M x S_1_	4	5.514	23.887***
	Error	20	0.231	
Stevioside–younger leaves	M	4	9.282	150.060***
	S_1_	1	4.042	65.350***
	M x S_1_	4	3.796	61.370***
	Error	20	0.062	
Reb A–older leaves	M	4	200.330	54.210***
	S_1_	1	46.620	12.615**
	M x S_1_	4	23.820	6.447**
	Error	20	3.700	
Reb A–younger leaves	M	4	104.007	277.15***
	S_1_	1	180.400	480.72***
	M x S_1_	4	8.336	22.21***
	Error	20	0.375	

^ns^ not significant

** significant at the 0.01 probability level

*** significant at the 0.001 probability level

M– 0, 5, 20, 100, 500 μM MEL

S– 0, 50, 150 mM NaCl

S_1_−0, 50 mM NaCl

### The effect of NaCl on germination (the second germination experiment)

The presence of NaCl in AG distinctly inhibited stevia seed germination (Fig 2B). An increase in NaCl concentration caused a significant (P < 0.001) reduction in the number of germinating seeds compared to the control (GC = 61%). At the lowest tested concentration of NaCl (50 mM), GC was 41%, and, at the highest NaCl concentration (200 mM), it was only 16%.

### The effect of MEL on seed germination under the conditions of NaCl (the third germination experiment)

For this experiment, two concentrations of NaCl (50 mM and 150 mM) were chosen. The seeds placed in AG with 50 mM NaCl began germinating on the second day of incubation, and all tested MEL concentrations improved germination compared to the control seeds (0MEL), among which GE and GC reached only 28% and 39%, respectively (Fig 2C). The most favorable effect on germination was observed for 20MEL (GE = 46%, GC = 61%) and 5MEL (GE = 44%, GC = 54%). However, the positive effect gradually decreased as MEL concentration was further increased. Quite a different situation was observed when seeds were germinated in AG with 150 mM of NaCl (Fig 2D). In that case, control seeds, (0MEL), showed the best germination (GE = GC = 24%), while MEL significantly (P < 0.05) decreased germination as its concentration increased; the GE and GC for 500MEL accounted for 5% and 6%, respectively. Two-way ANOVA showed the significant (P < 0.001) effects of both MEL and NaCl on stevia seed germination (Table 2).

### Seedling development

**Fig 3:**
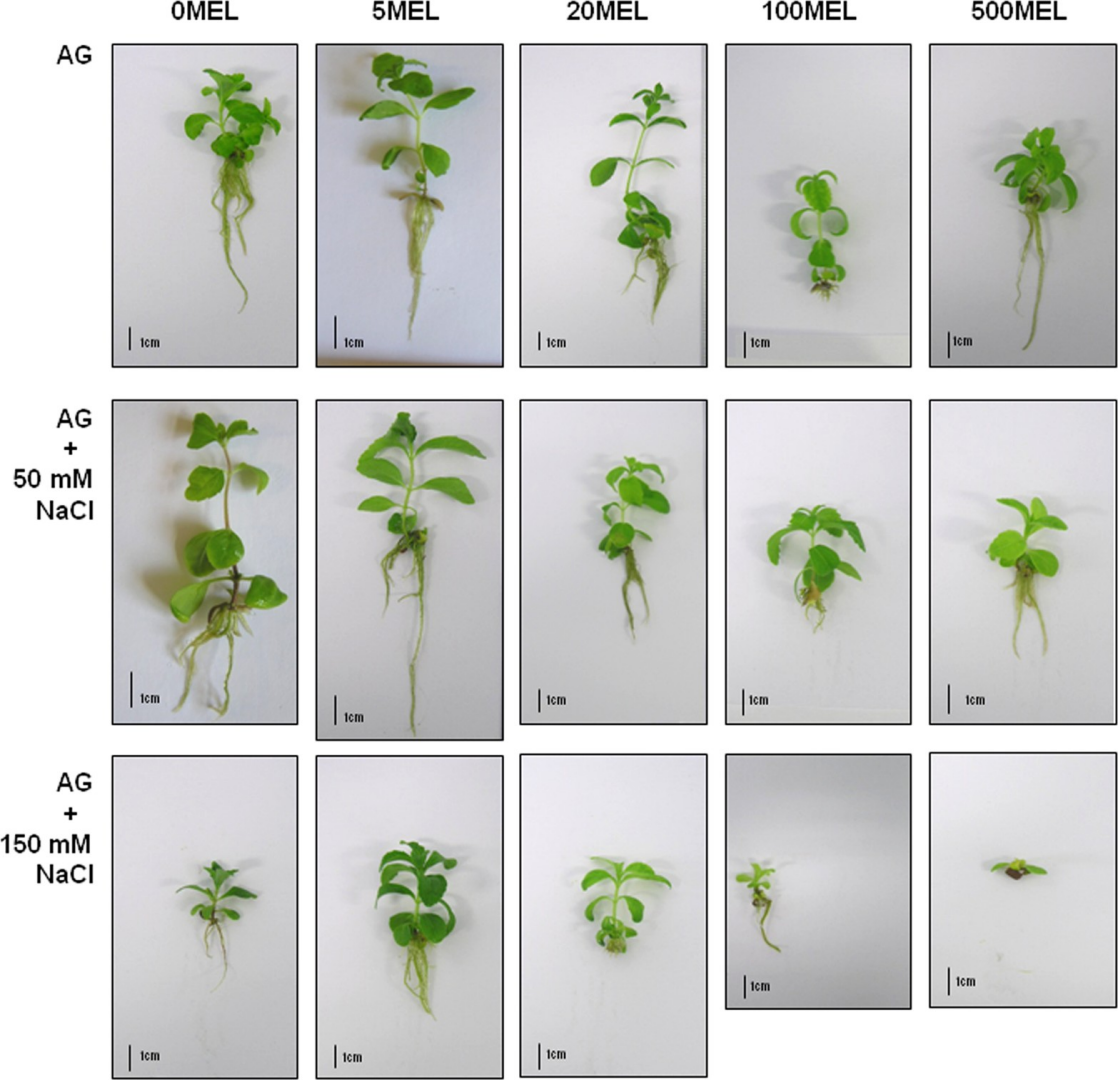
*Stevia rebaudiana* plantlets after four weeks growth in in vitro conditions on MS medium. Plantlets were obtained from seeds soaked with MEL and germinated on AG or on AG supplemented with 50 or 150 mM of NaCl.

Among seedlings obtained in the first germination experiment, 5MEL and 20MEL had the most beneficial effects on growth after four weeks in in vitro conditions (Fig 3, Table 3). Both fresh weight (FW) and stem length were the greatest and were significantly higher compared with the control (0MEL). On the other hand, the effect of MEL on leaf development was not observed. Regarding roots, large numbers of roots were observed in plantlets developed from seeds treated with 500 μM of MEL, but, compared with the control, the difference was not significant. Other MEL concentrations inhibited root development. Concerning root length, MEL inhibited growth as its concentration increased.

**Table 3:** Effects of MEL and NaCl on stevia growth in in vitro conditions. Melatonin were used for seed soaking, whilst NaCl were added to the agar gel (AG). Obtained seedlings were grown four weeks on MS medium. The means (n = 5) ±SD followed by different letters, are significantly different based on ANOVA followed by Duncan's test at P < 0.05.

MEL (μM)	NaCl (mM)	Fresh weight (g)	Shoot fresh weight (g)	Stem length (cm)	Leaves/plantlets number	Roots number	Root length (cm)
0 (control)	0	0.23 ± 0.07bc	0.10 ± 0.02b	2.24 ± 0.85c	9.2 ± 1.09a	11.0 ± 2.00a	4.54 ± 1.20a
5		0.35 ± 0.05ab	0.24 ± 0.04a	4.14 ± 0.56a	9.6 ± 0.00a	7.6 ± 1.14b	3.86 ± 1.02b
20		0.41 ± 0.19a	0.20 ± 0.06a	4.00 ± 0.46ab	10.8 ± 1.09a	7.4 ± 1.82b	3.78 ± 1.44b
100		0.21 ± 0.02c	0.14 ± 0.03b	3.22 ± 0.76b	9.6 ± 1.67a	6.0 ± 1.00b	0.74 ± 0.05d
500		0.20 ± 0.03c	0.12 ± 0.07b	2.12 ± 0.15c	9.2 ± 1.09a	12.2 ± 2.28a	2.90 ± 0.16c
0 (control)	50	0.76 ± 0.12a	0.53 ± 0.07a	3.06 ± 0.69a	8.8 ± 1.09ab	12.8 ± 2.28a	6.92 ± 0.71a
5		0.31 ± 0.05b	0.16 ± 0.04b	2.38 ± 0.83ab	9.6 ± 0.89a	11.0 ± 2.00ab	4.25 ± 2.13b
20		0.25 ± 0.02b	0.18 ± 0.01b	1.68 ± 0.26bc	7.2 ± 1.09b	9.2 ± 4.5ab	3.06 ± 0.29bc
100		0.14 ± 0.04c	0.10 ± 0.03c	1.96 ± 0.59bc	7.2 ± 1.09b	8.8 ± 1.09b	0.84 ± 0.31d
500		0.10 ± 0.05c	0.05 ± 0.02c	1.33 ± 0.61c	5.2 ± 1.79c	5.2 ± 1.64c	2.07 ± 0.60cd

In the third germination experiment, the greatest growth of plantlets was observed in the control group (0MEL) (Fig 3, Table 3). It can, therefore, be assumed that MEL-soaking and germination in the presence of 50 mM NaCl significantly (P < 0.01) reduces the mass and length of plantlets as MEL concentration increases (Table 2, Table 3). In NaCl conditions, MEL also inhibited root growth because the longest roots were observed in plants obtained from control seeds (0MEL), and, as MEL concentration increased, plantlets developed shorter and shorter roots. In the presence of 50 mM NaCl, MEL also exerted an inhibitory effect on root development, as, again, the largest numbers of roots were observed in plantlets obtained from control seeds (0MEL). Only treatment with 5MEL positively affected leaf development. Nevertheless, in this research, the plantlets developed from seeds germinated in AG containing NaCl at a concentration of 50 mM were characterized by good morphological parameters. However, the presence of 150 mM NaCl exerted an unfavorable effect on the further development of seedlings, which resulted in inhibited growth or even death (Fig 3). Because of their poor condition, such plantlets were excluded from further analyses.

### Steviol glycoside content

**Fig 4:**
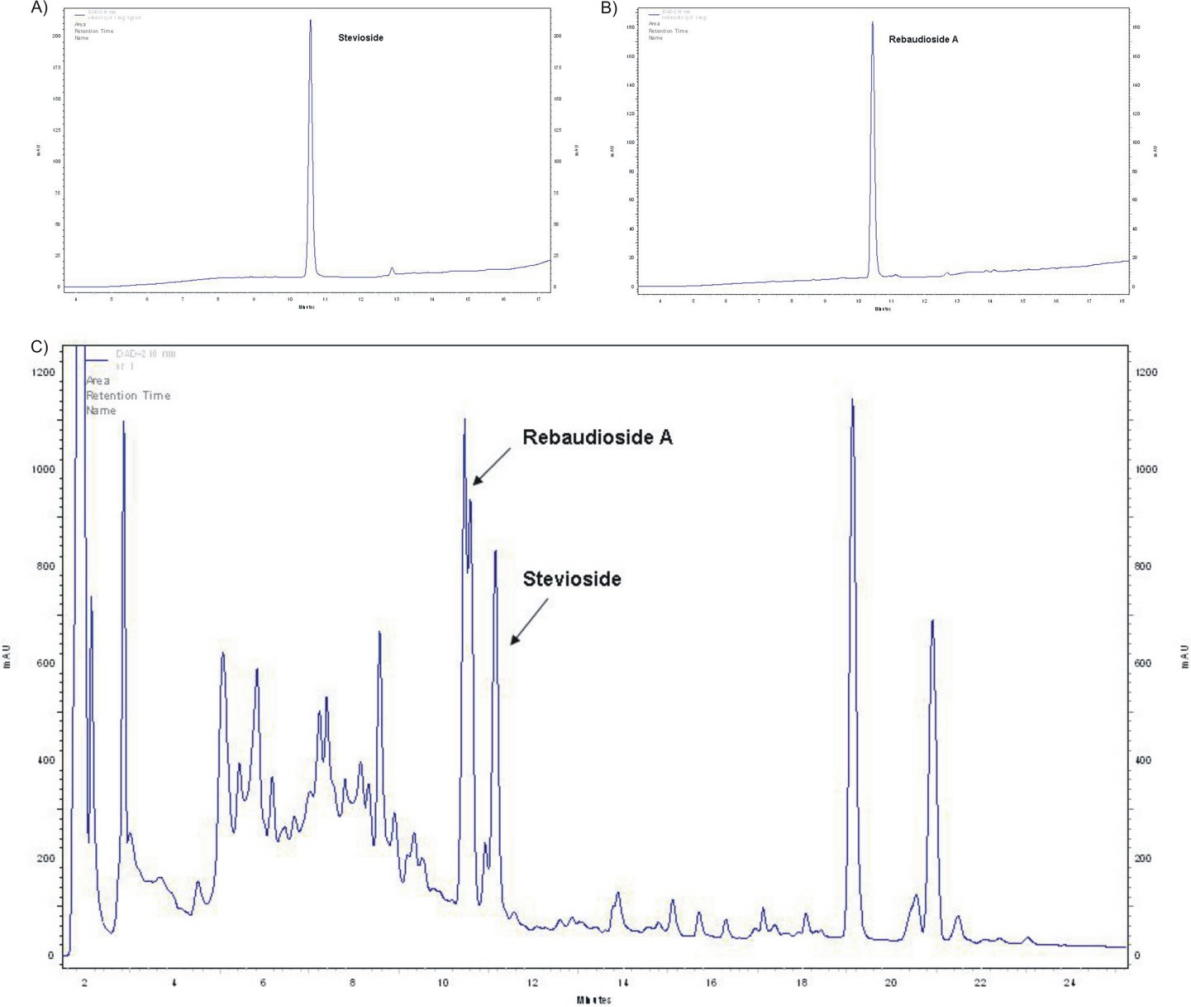
HPLC chromatograms of the standards: stevioside (A); rebaudioside A (B); HPLC chromatogram of the steviol glycosides fraction extract from *Stevia rebaudiana* leaves (older leaves, 0MEL, AG) (C).

**Fig 5:**
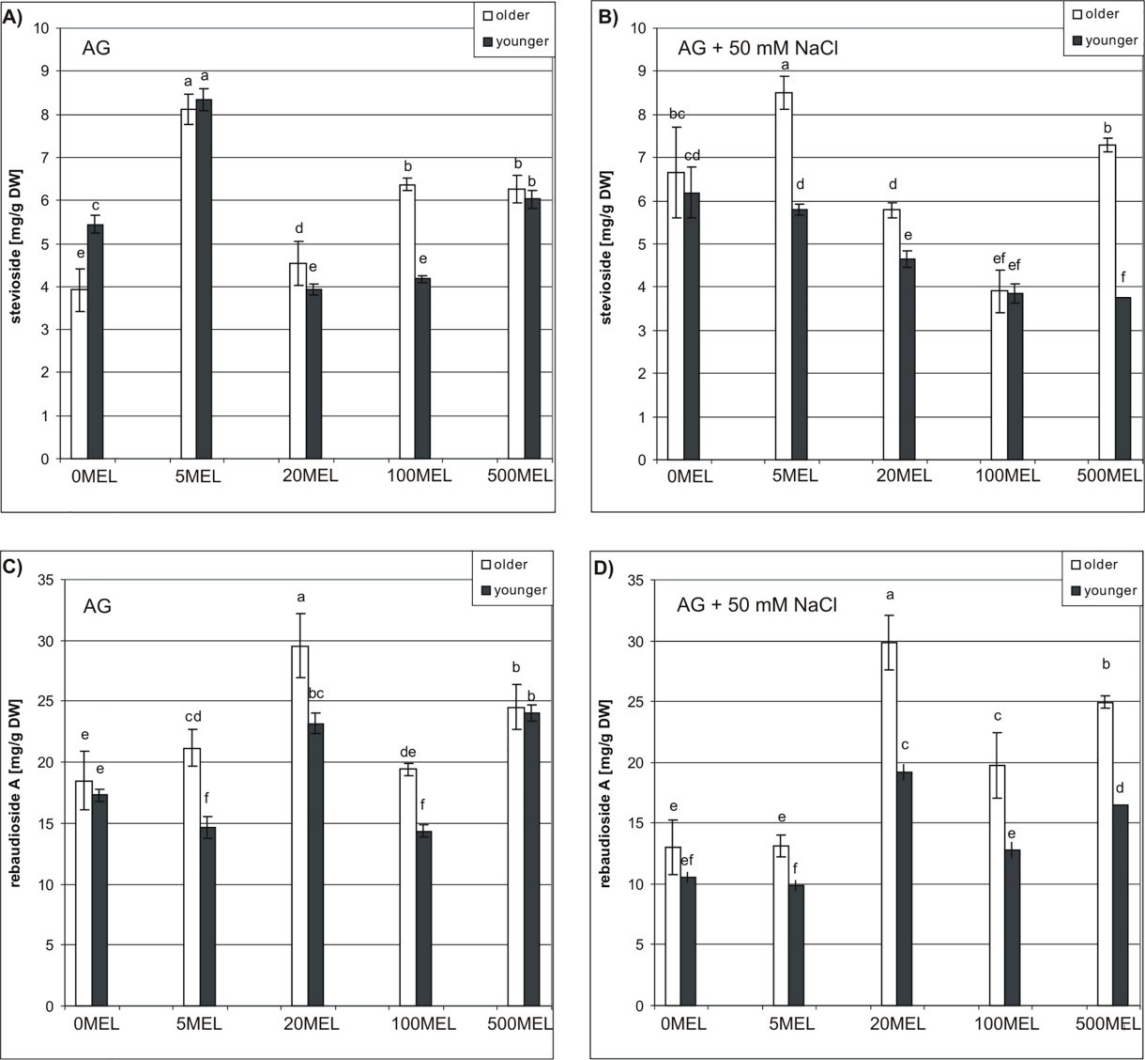
The content of stevioside (A, B) and rebaudioside A (C, D) in older and younger leaves of stevia. The plants were grown for 6 month under controlled conditions. The plants were obtained from MEL-soaked seeds germinated on AG (A, C) or on AG supplemented with 50 mM NaCl (B, D). The results are means of three replicates (n = 3) ±SD. DW–dry weight. Different letters indicate a significant difference at P < 0.05 according to ANOVA and Duncan’s test.

Stevioside and Reb A were identified by comparison with authentic compounds (Fig 4, S2 Fig, S3 Fig). Both SGs were detected in all samples; however, their concentrations varied dramatically according to MEL concentration and germination conditions (AG or AG + 50 mM NaCl) (Fig 5). Regarding older leaves of plants obtained from seeds germinated in AG, the highest stevioside content, 8.11 mg/g dry weight (DW), was observed in the 5MEL case. The positive effect of other MEL concentrations was also visible, as compared to the control group. In younger leaves, 5MEL had the most favorable effect on stevioside content as well, with 8.35 mg/g DW detected, compared to 5.44 mg/g DW in the younger leaves of control plants. This beneficial effect on the stevioside content of younger leaves was also shown for 500MEL (Fig 5A). In the presence of NaCl (AG + 50 mM NaCl), 5MEL and 500MEL favorably affected the stevioside content of older leaves, while, in younger leaves, all MEL concentrations distinctly reduced stevioside content (Fig 5B). This effect was more and more pronounced as MEL concentrations increased.

All tested MEL concentrations favorably affected the Reb A content of older leaves, and the highest level of this compound was found for 20MEL (Fig 5C)—approximately 1.6 times higher than the content in older leaves of control plants. 500MEL and 5MEL also caused a significant increase in Reb A content compared with control plants. In younger leaves, the highest Reb A content was found in 20MEL and 500MEL treatments. However, the other two concentrations of MEL (5 MEL and 100MEL) reduced Reb A content compared with the control (Fig 5C). Under NaCl conditions, Reb A content was highest in the older leaves of plants obtained from seeds treated with 20MEL (Fig 5D). 500MEL and 100MEL also exerted favorable effects. Regarding younger leaves, the exact same relationship was found. It is, however, worth noting that, regardless of MEL concentration and seed germination conditions (AG or AG + 50 mM NaCl), higher amounts of Reb A were accumulated in older leaves than in younger leaves.

### Gene expression analysis

**Fig 6:**
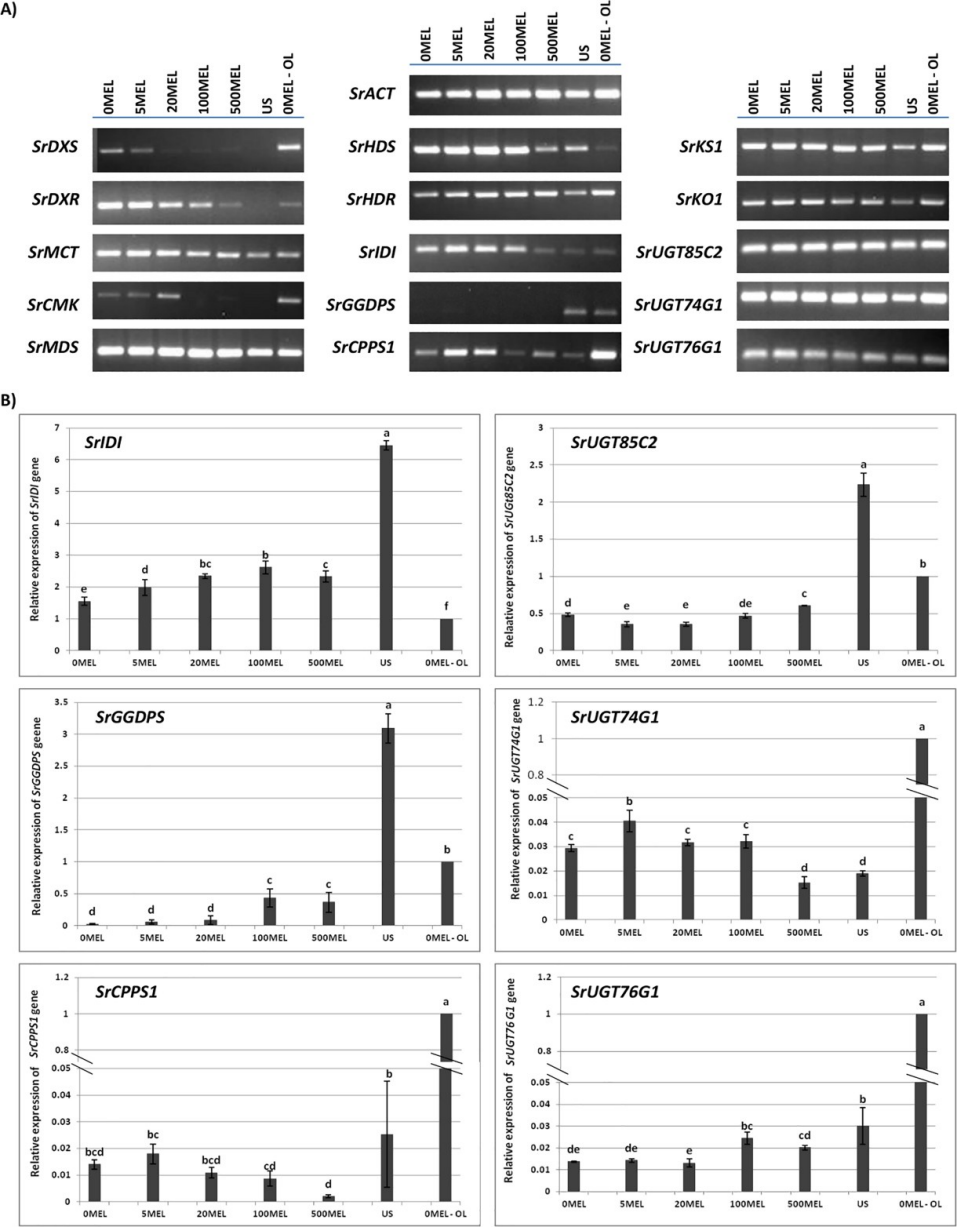
RT-PCR (A) and qPCR (B) results for steviol glycoside biosynthetic pathway genes in stevia seeds. The seeds were soaked in aqueous solutions of MEL (0MEL, 5MEL, 20MEL, 100MEL and 500MEL). Dry untreated seeds (US) were also used for comparison. RNA for each replication was extracted from 20 seeds. The relative expression of older leaves of control plants (0MEL—obtained from seeds incubated in water before germination; 0MEL-OL) was set at 1.0. The expression was normalized to the expression of the *S*. *rebaudiana actin* gene (*SrACT*). The results of qPCR are the means of three biological replicates ±SD (n = 3). Different letters indicate a significant difference at P < 0.05 according to ANOVA and Duncan’s test.

The seeds incubated in MEL before germination expressed the genes involved in the SG biosynthesis pathway (Fig 6A). Only the transcription for the *SrGGDPS* gene was not observed in any seed sample after RT-PCR analysis. On the other hand, the most variable expression profiles were observed for genes involved in the first step of SG biosynthesis—i.e., the MEP pathway. qPCR showed that the incubation of seeds, before sowing, in water or MEL solutions, significantly decreases the expression of the following genes: *SrIDI*, *SrGGDPS*, *SrCPPS1*, *SrUGT85C2* and *SrUGT76G1* (Fig 6B). It also increases the expression of the *SrUGT74G1* gene but only at the lowest MEL concentration. It is also worth noting, that, the relative expression of the *SrIDI* gene was at least 1.5 times higher in seed samples compared with leaves (Fig 6B).

**Fig 7:**
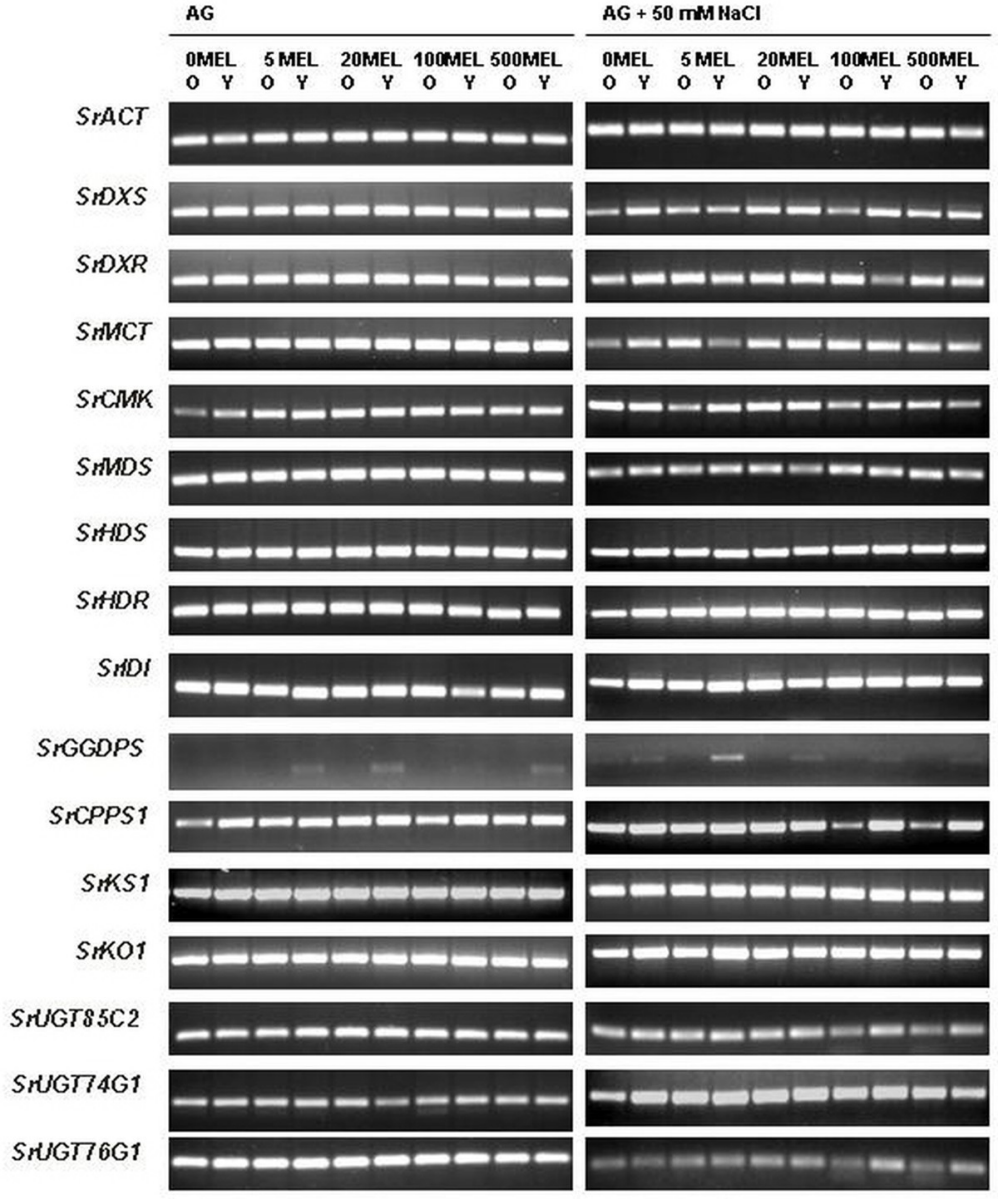
RT-PCR results of steviol glycoside biosynthesis pathway genes in older (O) and younger (Y) leaves of stevia. The plants were grown for 6 months under controlled conditions. The plants were obtained from MEL-soaked seeds, germinated on AG or on AG supplemented with 50 mM NaCl. *S*. *rebaudiana actin* gene (*SrACT*) was used as reference gene.

**Fig 8:**
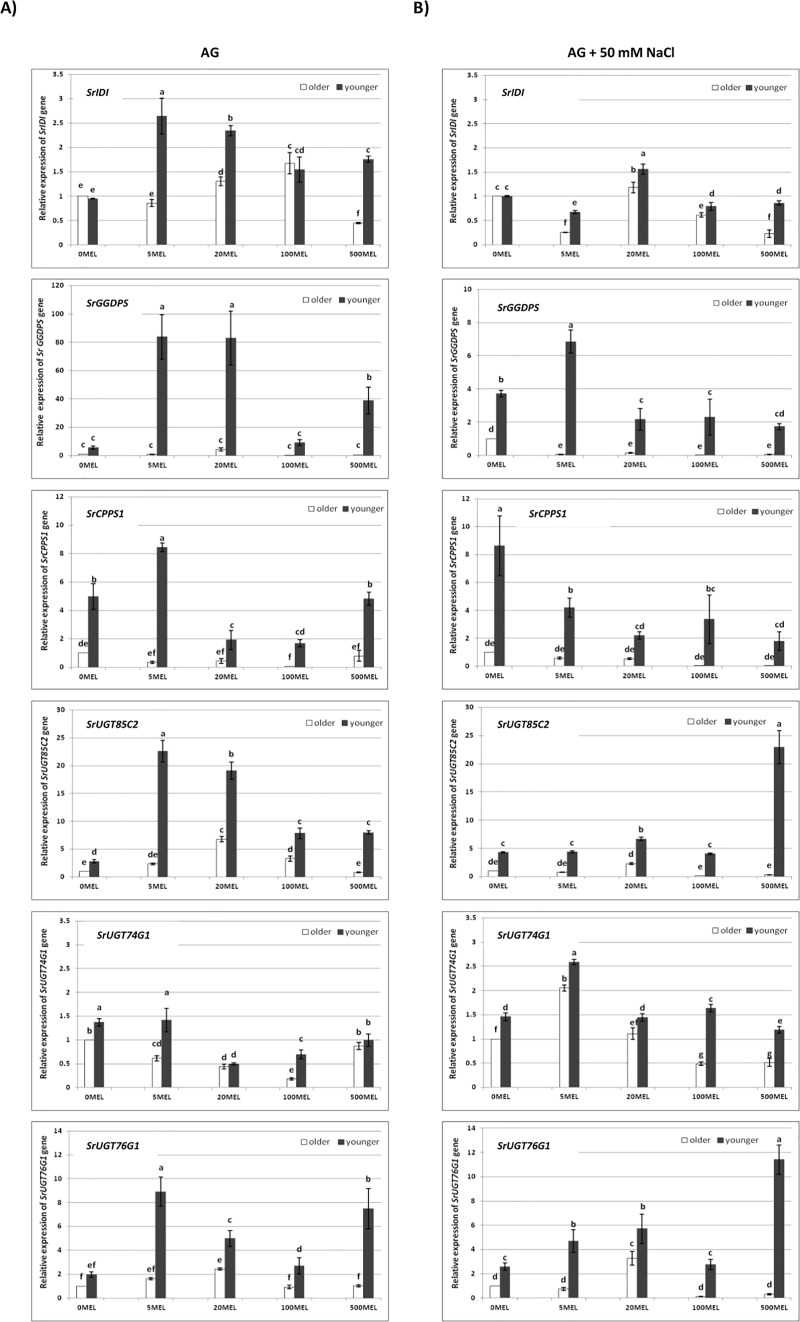
qPCR results for the relative expression of steviol glycoside biosynthesis pathway genes in stevia leaves. The plants were grown for 6 months under controlled conditions. The plants were obtained from MEL-soaked seeds, germinated on AG or on AG supplemented with 50 mM NaCl. The relative expression of older leaves of control plants (obtained from seeds incubated in water before germination; 0MEL) was set at 1.0. The expression was normalized to the expression of the *S*. *rebaudiana actin* gene (*SrACT*). The results of qPCR are the means of three biological replicates ±SD (n = 3). Different letters indicate a significant difference at P < 0.05 according to ANOVA and Duncan’s test.

In leaves, the transcription patterns of many of the tested SG genes also varied in ways associated with the type of leaves and seed germination conditions (MEL and NaCl) (Fig 7). In the leaves of plants obtained from seeds germinated without NaCl, the expression pattern of the *SrGGDPS* gene showed the most visible differences; in the leaves of control plants, no transcript was observed, just as in the older leaves under MEL treatment. The transcript was detected only in younger leaves under MEL treatment, but its level varied. The results of qPCR confirmed the relationships between the transcript levels observed in the gel after electrophoresis, where the highest gene expression was found in the leaves of plants treated with 5MEL and 20MEL (Fig 8). Nevertheless, qPCR, as a more sensitive method, allowed observations of trace amounts of the *SrGGDPS* gene transcript in leaf samples from control plants and in older leaves under MEL treatment. However, the expression of this gene was strongly correlated with Reb A accumulation just in older leaves (Table 4). The qPCR analysis for other selected genes also showed a higher level of transcript accumulation in younger leaves than in older ones. In younger leaves, upregulation of *SrIDI*, *SrCPPS1*, and all three UDP-glycosyltransferase genes was observed, and the highest level of their transcripts was found under the influence of 5MEL. The analysis of correlations showed a strong relationship between the level of the *SrCPPS1* gene transcript and stevioside accumulation. Also, under the influence of 20MEL, the expression of *SrIDI*, *SrUGT85C2*, and *SrUGT76G1* in younger leaves was higher compared with the control. For those genes, the highest correlation with stevioside content was observed in younger leaves, and, with the RebA content, in older leaves (Table 4).

**Table 4:** The coefficient of correlation between the stevioside and rabaudioside A (Reb A) contents in stevia leaves and the expression of their biosynthesis genes. Correlation coefficients were calculated using Microsoft Excel by applying the Pearson formula.

Steviol glycoside	*Gene*	*SrIDI*		*SrGGDPS*		*SrCPPS1*		*SrUGT85C2*		*SrUGT74G1*		*SrUGT76G1*	
	Type of leaf	older leaves	younger leaves	older leaves	younger leaves	older leaves	younger leaves	older leaves	younger leaves	older leaves	younger leaves	older leaves	younger leaves
**AG**													
**Stevioside**	older leaves	-0.18		-0.42		- 0.44		- 0.22		- 0.28		- 0.10	
	younger leaves		0.39		0.35		0.95		0.38		0.78		0.69
**Reb A**	older leaves	-0.09		0.80		0.01		0.63		-0.16	-0.41	0.75	0.21
	younger leaves		0.10		0.27		-0.28		-0.01				
**AG + 50mM NaCl**													
**Stevioside**	older leaves	- 0.43		0.09		0.35		0.05		0.65		-0.048	
	younger leaves		-0.07		0.70		0.77		-0.54		0.46		-0.51
**Reb A**	older leaves	0.26		- 0.46		- 0.46		0.44		-0.43		0.52	
	younger leaves		0.72		-0.69		- 0.62		0.48		- 0.62		0.55

In the leaves of plants obtained from seeds germinated on AG with 50 mM of NaCl, the RT-PCR patterns of *SrMDS*, *SrHDS*, and *SrKS1* genes showed no visible changes (Fig 7). Conversely, *SrGGDPS* was significantly affected by different MEL concentrations: its transcript was observed in both types of leaves of the control plants; however, when plants were obtained from MEL-soaked seeds, this transcript was observed only in younger leaves. The qPCR analysis results showed that the transcription of *SrGGDPS* gene in younger leaves of 5MEL-treated plants was upregulated, while, in older leaves, it was downregulated (Fig 8). The expression of *SrCPPS1* was also lower in older leaves compared to younger ones, in which it was significantly downregulated by MEL. However, the expression of both genes indicated a positive correlation with stevioside content and a negative correlation with Reb A content in younger leaves (Table 4). Regarding *SrIDI*, there were no visible differences between older and younger leaves of the control plants; however, qPCR indicated that, under the influence of 20MEL, the expression of *SrIDI* in both types of leaves was significantly upregulated in contrast to other MEL concentrations. Additionally, for all three genes encoding UDP-glycosyltransferases, expression levels were higher in younger leaves than in older ones. For *SrUGT85C2* and *SrUGT76G1*, the highest transcript accumulation was noted under the influence of 500MEL, while the highest *SrUGT74G1* transcript accumulation was observed when 5MEL was used. The expression of the *SrUGT74G1* gene was also most strongly correlated with SG content (Table 4).

## Discussion

Some evidence suggests that exogenous MEL might improve seed germination and seedling growth–both of which are critical stages of development determining plant quality [6, 9, 33]. The results presented in this research have indicated that MEL, at lower concentrations, stimulates germination, but, at higher concentrations (100 and 500 μM), it inhibits germination. This agrees with the authors’ previous research [27], in which MEL was added to the AG. Recently, for *Gossypium hirsutum*, similar relationships were described [34]. This confirms that the actions of MEL depend on its concentrations [35, 36]. The researchers’ work on stevia has shown that how MEL is applied to seeds does not significantly affect germination, but MEL concentrations do.

In this research, stevia seeds were able to germinate under salt conditions, although the highest GC was observed in the total absence of NaCl. This agrees with previous reports concerning stevia [37] and other species [38, 39]. As Zhang et al. [40] find, NaCl stress may inhibit ABA catabolism and GA_3_ biosynthesis and, consequently, delay germination.

MEL exerts an alleviatory effect on seed germination under salinity [38, 40]. However, in this research, the effect was visible only in the case of 50 mM of NaCl. When a higher concentration of NaCl was used (150 mM), MEL inhibited germination. However, in the research of Zhang et al. [40], MEL, at lower concentrations (0.1 μM—100 μM), reduced the inhibitory effect of 150 mM NaCl. Negative effects were observed only for higher MEL concentrations (500 μM). Zhang et al. [40] also indicate that MEL, at lower concentrations, relieves the inhibitory effect of high salinity on germination by upregulating antioxidant enzymes and downregulating pro-oxidant enzymes, as well as by acting as a signaling molecule stimulating both ABA catabolism and GA biosynthesis.

The results of the present study also suggest that MEL regulates stevia seedling development. A lower MEL concentration improves seedling growth, while a higher concentration attenuates, or even prevents, seedling development. Posmyk et al. [33] and Zhang et al. [40] have also noted that, at the highest concentrations, MEL acts negatively on plants.

Applying MEL before germination under salinity conditions did not improve seedling growth, and some morphological parameters were worsened compared with the control. This was especially evident for higher MEL concentrations. In the work of Zeng et al. [19], only the FW and DW of shoots and roots from *Brassica napus* seedlings were significantly higher under MEL and NaCl conditions, while shoot and root lengths and leaf area were reduced. Some reports [8, 41] suggest that, under salt stress, the optimum concentration of exogenous melatonin is different for various plants species. It is likely that for different species the optimal method of melatonin application is also different.

These results constitute proof, for the first time, of the stimulating effect low MEL concentrations have on the biosynthesis of SGs in stevia. To the best of the authors’ knowledge, such studies on stevia have not been previously performed. Other research has indicated that MEL, at 5 μM, enhances the biosynthesis of galanthamine in in vitro cultures of *L*. *aestivum* [21]. As the present work has shown, the negative effect of NaCl on SG biosynthesis was alleviated by applying MEL in specific doses. There are no reports on using MEL as a potential stimulator of SG biosynthesis under salt stress conditions. Only studies concerning the accumulation of SGs under salt stress have been performed [42, 43].

The *SrGGDPS* gene was the most differentially expressed among all tested genes. It was upregulated under MEL treatment in younger leaves, regardless of the presence of NaCl. This might suggest an important role of MEL in the MEP steps of the SG biosynthesis in stevia leaves. In seeds the *SrGGDPS* gene was not expressed. Kumar et al. [30] have found that the expression of the *SrGGDPS* gene is also higher in younger leaves compared to older. Geranylgeranyl diphosphate (GGDP) is synthesized at the endpoint of the MEP pathway and serves as an intermediary for the biosynthesis of several isoprenoids [44]. In stevia, GGDP is converted into steviol, and then different SGs are formed [30, 45]. Ghaheri et al. [46] and Nasrullah et al. [47] also suggest that different expressions of the genes involved in the late stages of SG biosynthesis relate to leaf maturation. On the other hand, the stable expression of MEP pathway genes suggests their important role in plant growth and development, since many compounds, such as chlorophylls and diterpenoids, are synthesized in the MEP pathway. Environmental stress is also involved in regulating the expression of SG genes [48, 49]. Fallah et al. [43] have determined that the expressions of *SrUGT74G1* and *SrUGT76G1* are downregulated by NaCl. In the present experiments, NaCl also modulated the expression of these genes but particularly in combination with MEL.

## Conclusions

This study demonstrates the positive effect soaking seeds in 5 μM and 20 μM of MEL had on stevia germination in normal conditions and under low salinity stress. Additionally, lower MEL concentrations promoted the biosynthesis of stevioside and Reb A, alleviating salt stress. SG concentration correlated with the expression of SG biosynthesis pathway genes in leaves, and the authors have also shown, for the first time, the expression of SG biosynthesis genes in stevia seeds. However, further studies must be conducted to provide more molecular and genetic evidence to support the mechanisms of MEL-induced salt stress tolerance in stevia.

## Supporting information

S1 Fig Melting curves of qPCR products. (TIF)

S2 Fig HPLC trace and UV spectra of stevia extract with the addition of the rebaudioside A internal standard (A) and without the standard compound (B).
(TIF)

S3 Fig LC-MS confirmation of the presence of stevioside (S3A Fig) and rebaudioside A (S3B Fig) in the sample (older leaves, 0MEL).
(TIF)

S4 Fig PCR efficiency test for the *SrGGDPS* primers. The standard plot (A) was based on a series of five 2-fold cDNA dilutions, starting from 0.25x (sample: older leaves, 0MEL, AG + 50 mM NaCl). PCR was carried out in the 7500 Fast Real Time PCR System (Applied Biosystems) using 2X Fast SYBR Green Master Mix. The reaction mixture contained 2.5 μl of diluted cDNA. Additionally, the qPCR products from serial dilutions were examined by electrophoresis in a standard agarose gel (1.5%) (B). NTC–no template control, M–GeneRuler 100 bp Plus DNA Ladder (Thermo Scientific).
(TIF)

S1 Raw images (PDF)

S2 Raw Images (PDF)

S3 Raw Images (PDF)

S4 Raw Images (PDF)

S1 Data (XLS)
