# Supplementary figures and images for: Melatonin promotes seed germination under salinity and enhances the biosynthesis of steviol glycosides in Stevia rebaudiana Bertoni leaves
Source: PLoS One. 2020 Mar 27;15(3):e0230755. doi: 10.1371/journal.pone.0230755 (PMC7100979; doi:10.1371/journal.pone.0230755)

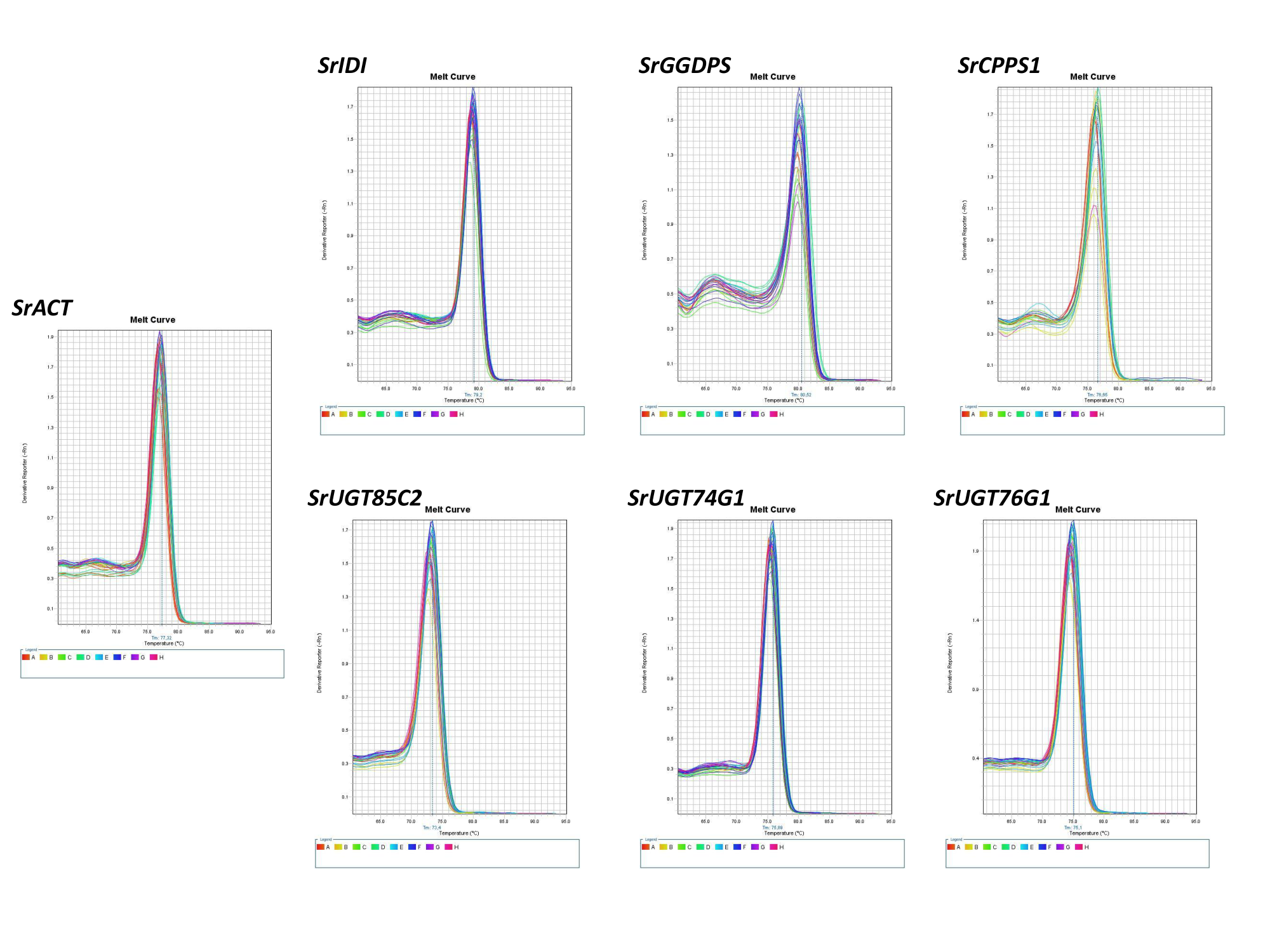

Supplement: S1 Fig — (TIF) [file pone.0230755.s001.tif]

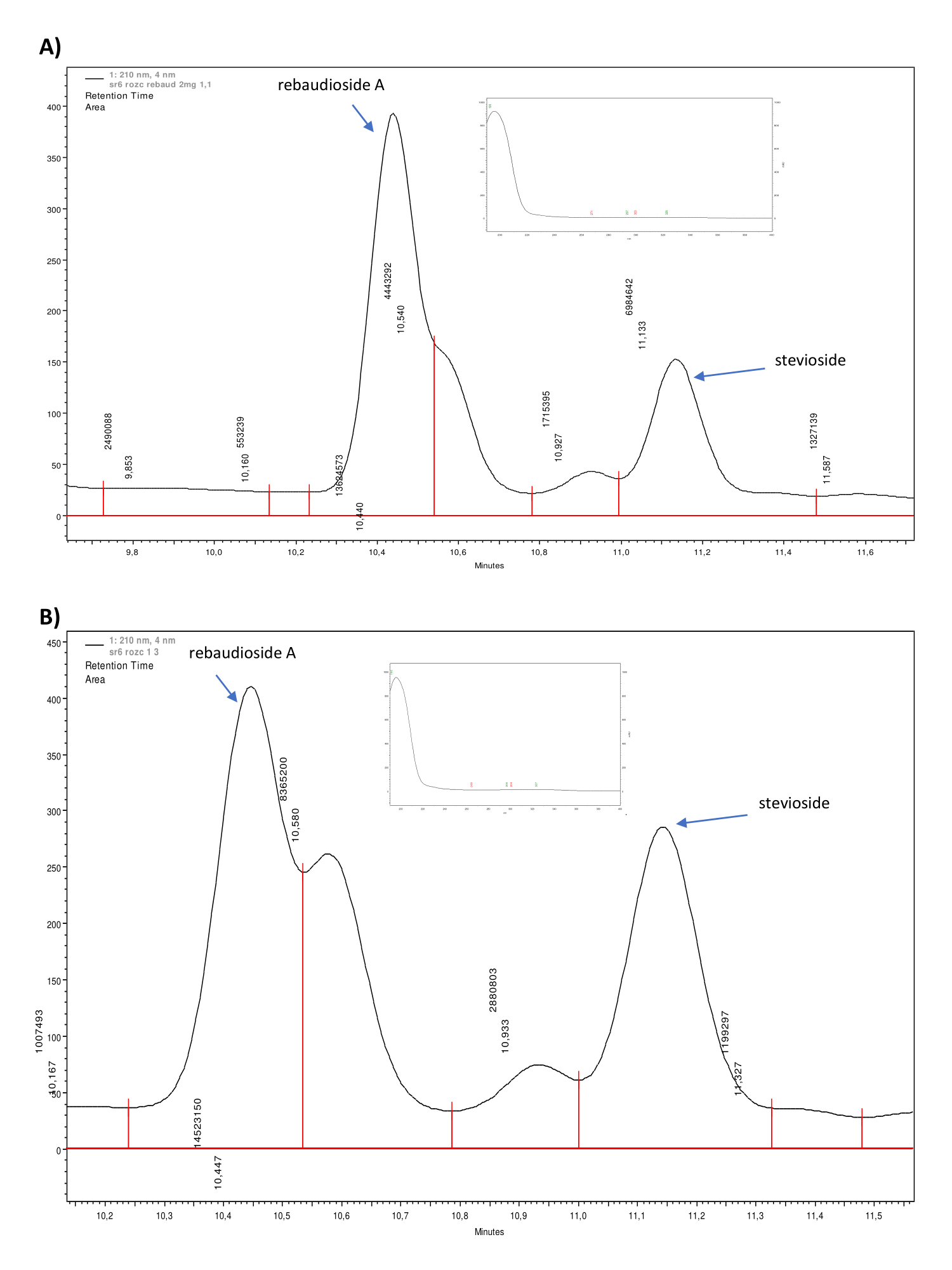

Supplement: S2 Fig — HPLC trace and UV spectra of stevia extract with the addition of the rebaudioside A internal standard (A) and without the standard compound (B). (TIF) [file pone.0230755.s002.tif]

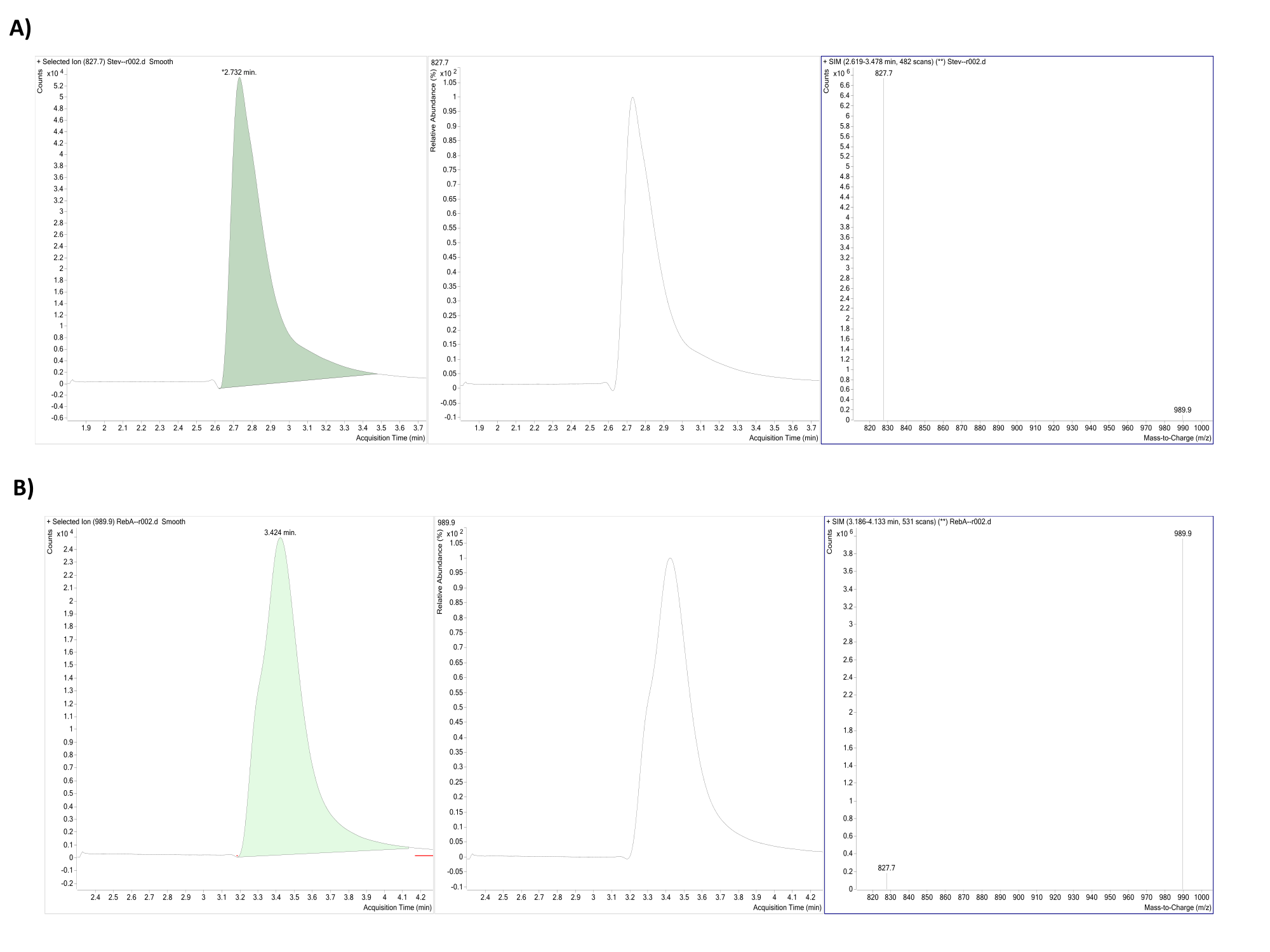

Supplement: S3 Fig — LC-MS confirmation of the presence of stevioside (S3A Fig) and rebaudioside A (S3B Fig) in the sample (older leaves, 0MEL). (TIF) [file pone.0230755.s003.tif]

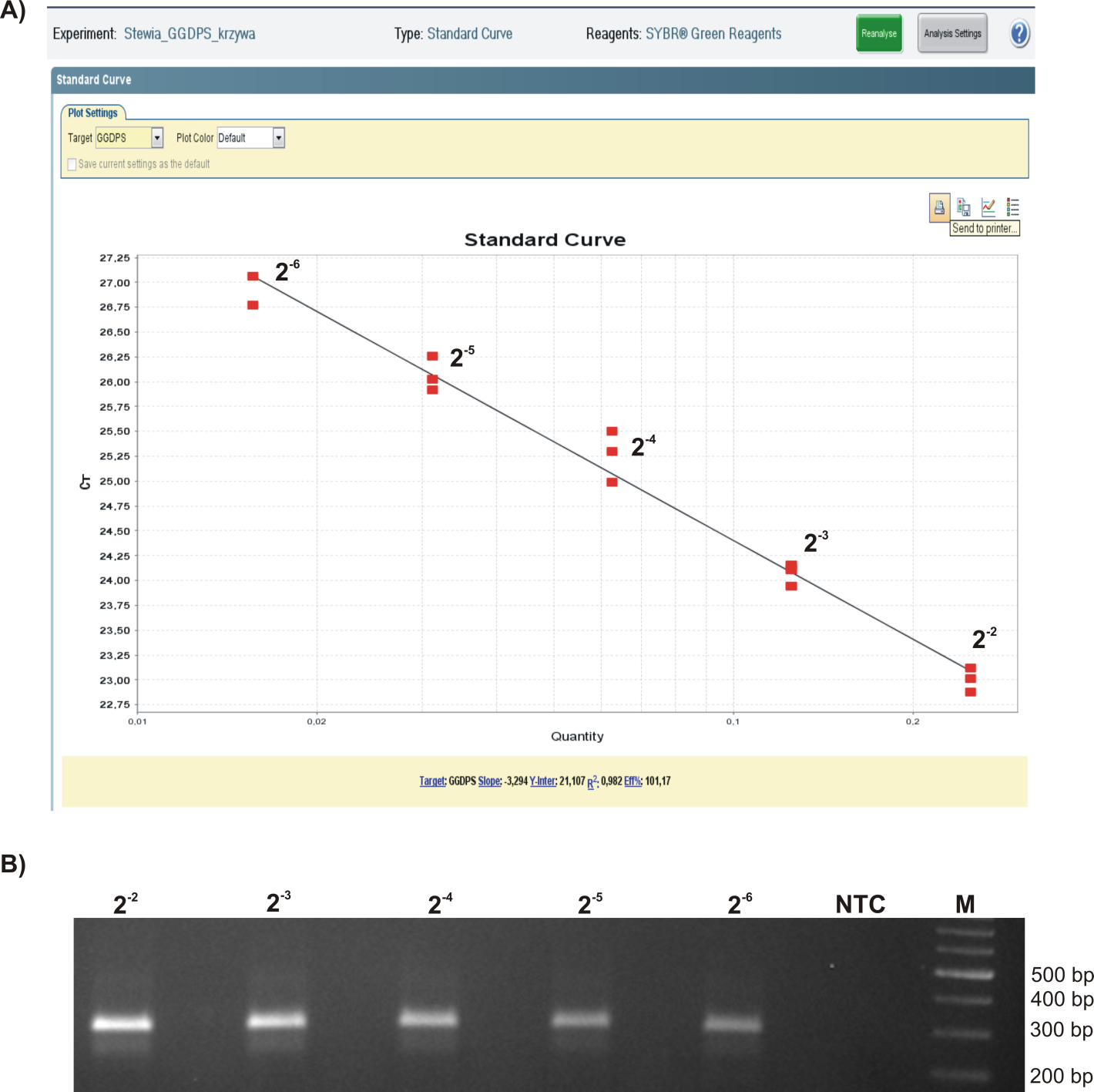

Supplement: S4 Fig — The standard plot (A) was based on a series of five 2-fold cDNA dilutions, starting from 0.25x (sample: older leaves, 0MEL, AG + 50 mM NaCl). PCR was carried out in the 7500 Fast Real Time PCR System (Applied Biosystems) using 2X Fast SYBR Green Master Mix. The reaction mixture contained 2.5 μl of diluted cDNA. Additionally, the qPCR products from serial dilutions were examined by electrophoresis in a standard agarose gel (1.5%) (B). NTC–no template control, M–GeneRuler 100 bp Plus DNA Ladder (Thermo Scientific). (TIF) [file pone.0230755.s004.TIF]
