# Supplementary material for: Melatonin promotes seed germination under salinity and enhances the biosynthesis of steviol glycosides in Stevia rebaudiana Bertoni leaves
Source: PLoS One. 2020 Mar 27;15(3):e0230755. doi: 10.1371/journal.pone.0230755 (PMC7100979; doi:10.1371/journal.pone.0230755)
Supplement: S1 Raw images — (PDF) [file pone.0230755.s005.pdf]

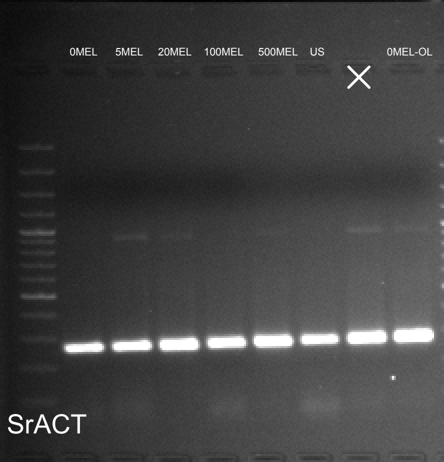
0MEL
5MEL
20MEL
100MEL
500MEL
US
0MEL-OL
SrACT

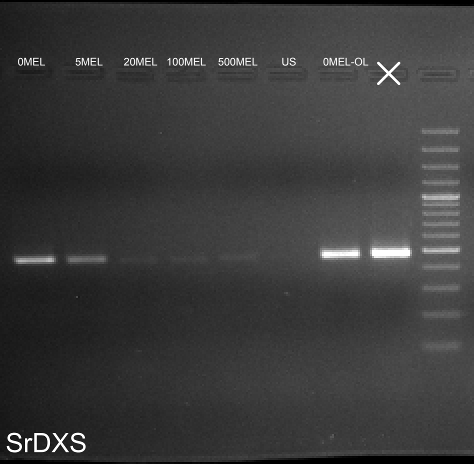
0MEL
5MEL
20MEL
100MEL
500MEL
US
0MEL-OL
SrDXS

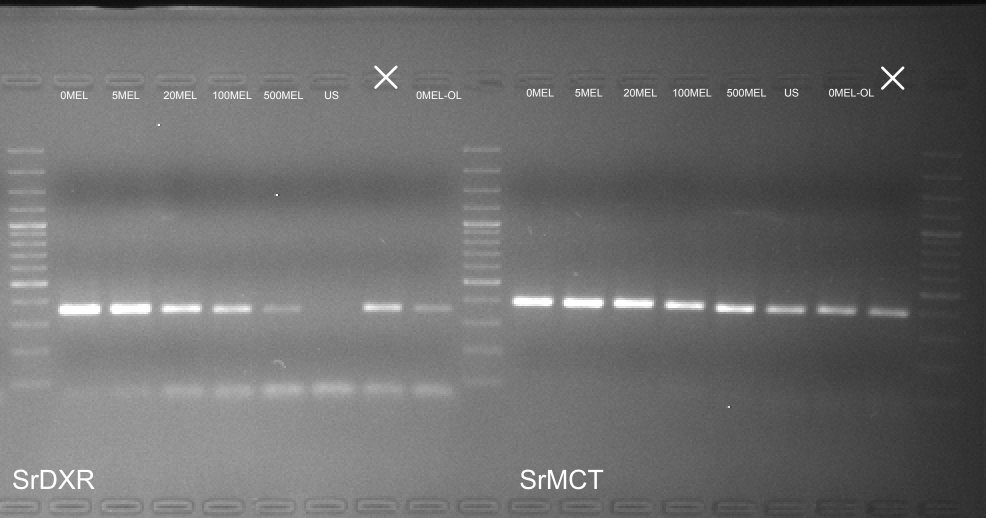
0MEL
5MEL
20MEL
100MEL
500MEL
US
0MEL-OL
0MEL
5MEL
20MEL
100MEL
500MEL
US
0MEL-OL
SrDXR
SrMCT

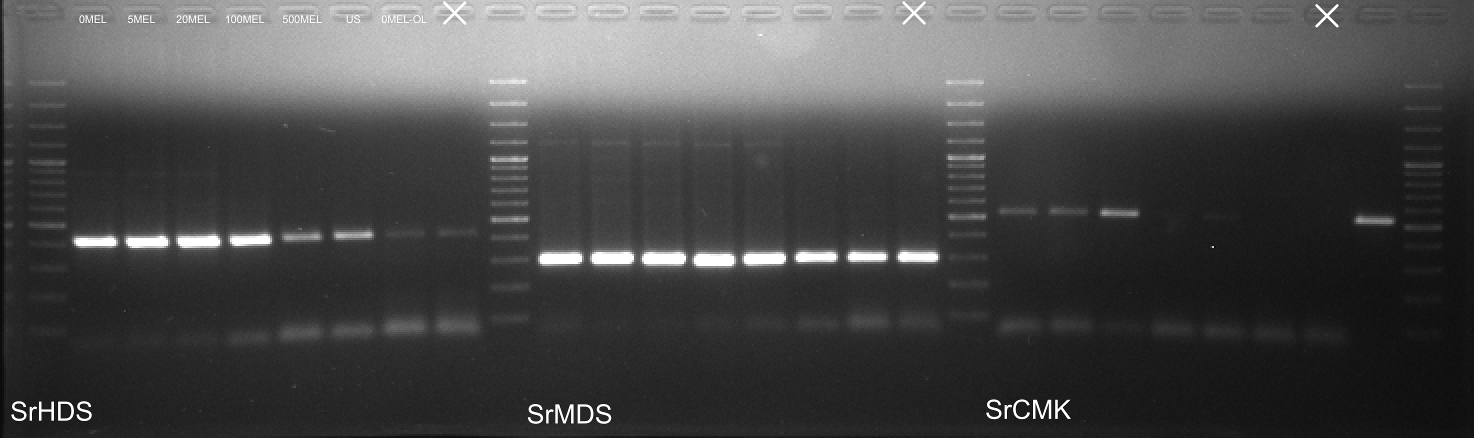
0MEL
5MEL
20MEL
100MEL
500MEL
US
0MEL-OL
SrHDS
SrMDS
SrCMK

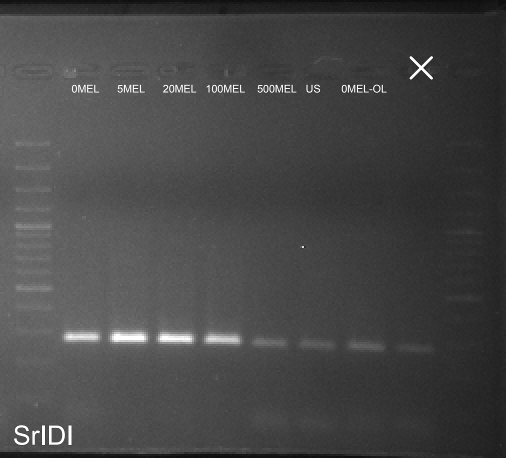
0MEL
5MEL
20MEL
100MEL
500MEL
US
0MEL-OL
SrIDI

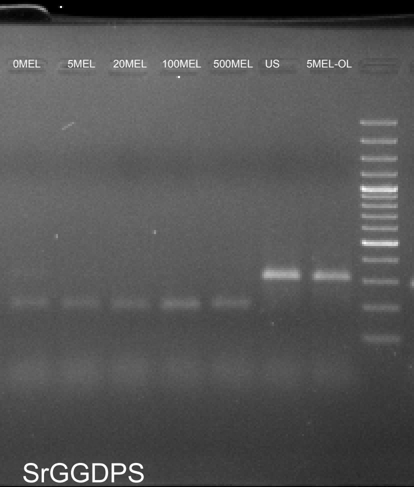
0MEL
5MEL
20MEL
100MEL
500MEL
US
5MEL-OL
SrGGDPS

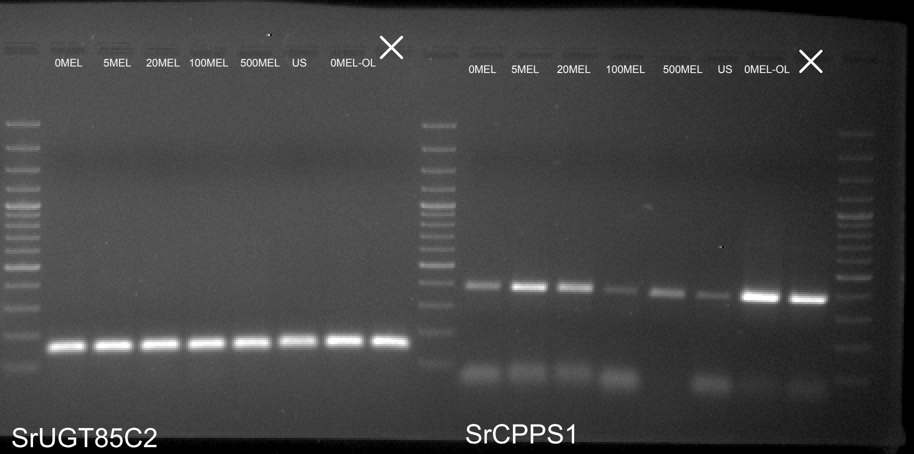
0MEL
5MEL
20MEL
100MEL
500MEL
US
0MEL-OL
0MEL
5MEL
20MEL
100MEL
500MEL
US
0MEL-OL
SrUGT85C2
SrCPPS1

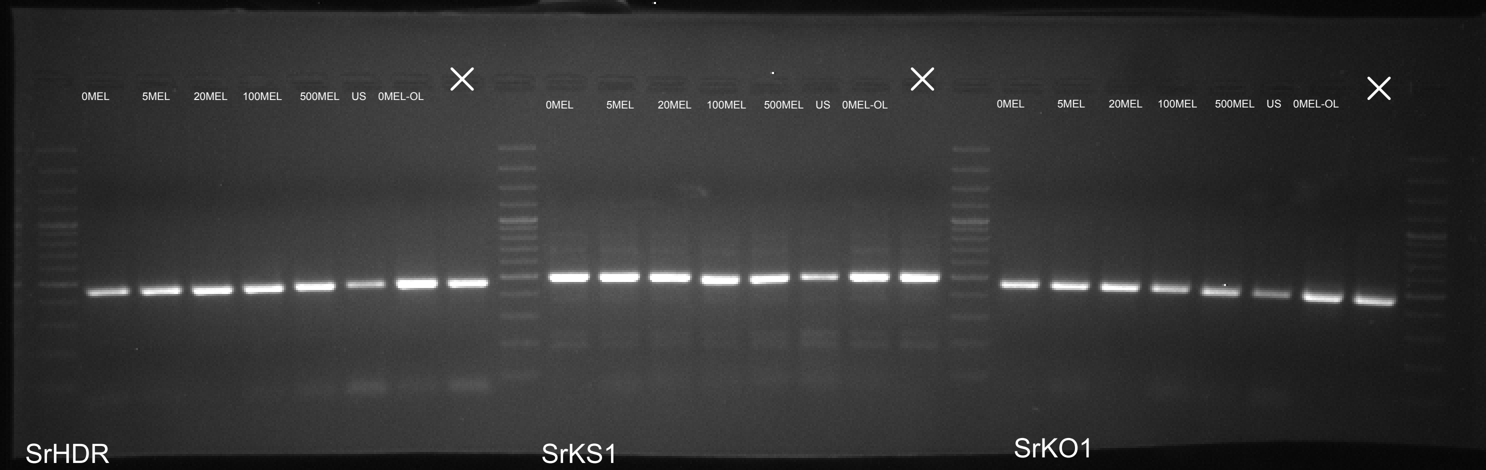
0MEL
5MEL
20MEL
100MEL
500MEL
US
0MEL-OL
0MEL
5MEL
20MEL
100MEL
500MEL
US
0MEL-OL
0MEL
5MEL
20MEL
100MEL
500MEL
US
0MEL-OL
SrHDR
SrKS1
SrKO1

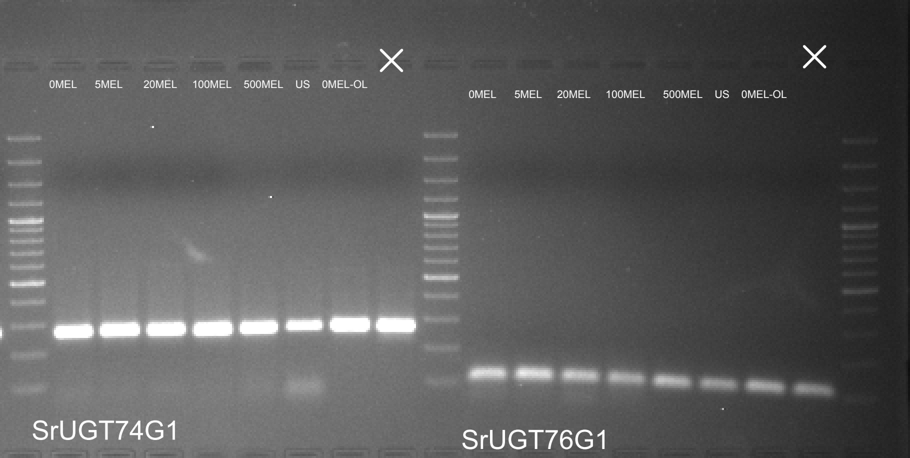
0MEL
5MEL
20MEL
100MEL
500MEL
US
0MEL-OL
0MEL
5MEL
20MEL
100MEL
500MEL
US
0MEL-OL
SrUGT74G1
SrUGT76G1
