# Supplementary material for: Melatonin promotes seed germination under salinity and enhances the biosynthesis of steviol glycosides in Stevia rebaudiana Bertoni leaves
Source: PLoS One. 2020 Mar 27;15(3):e0230755. doi: 10.1371/journal.pone.0230755 (PMC7100979; doi:10.1371/journal.pone.0230755)
Supplement: S3 Raw Images — (PDF) [file pone.0230755.s007.pdf]

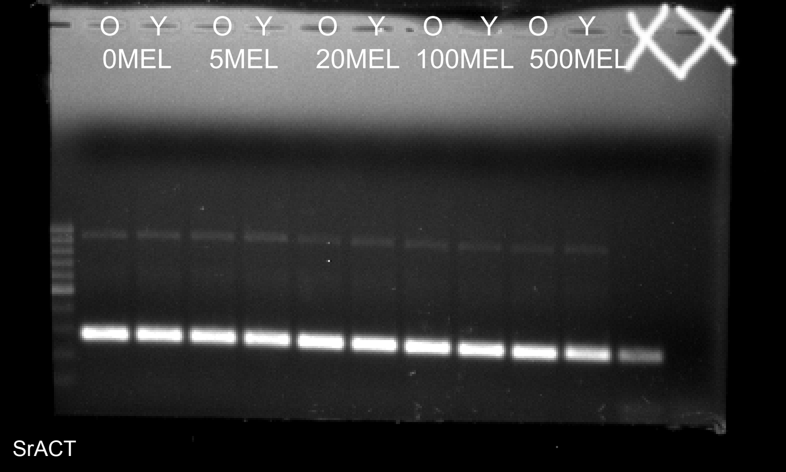
O Y O Y O Y O Y O Y
0MEL 5MEL 20MEL 100MEL 500MEL
SrACT

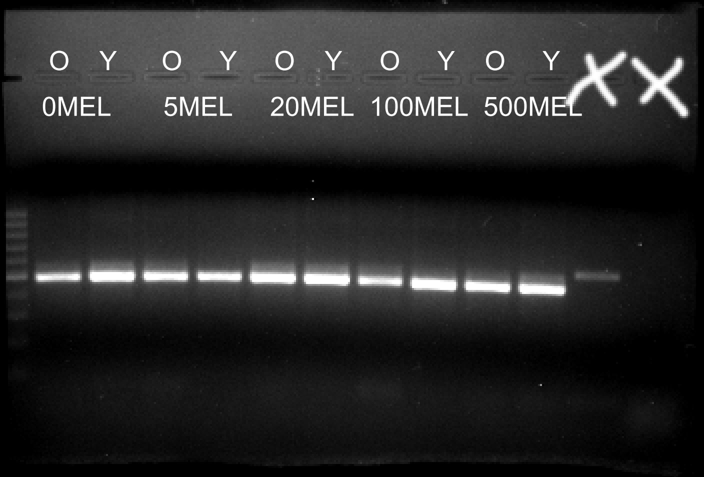

SrDXS

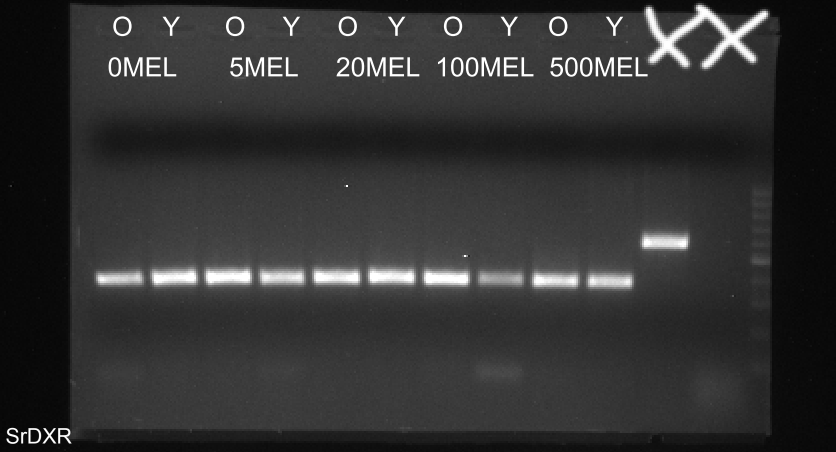
O Y O Y O Y O Y O Y
0MEL 5MEL 20MEL 100MEL 500MEL
SrDXR

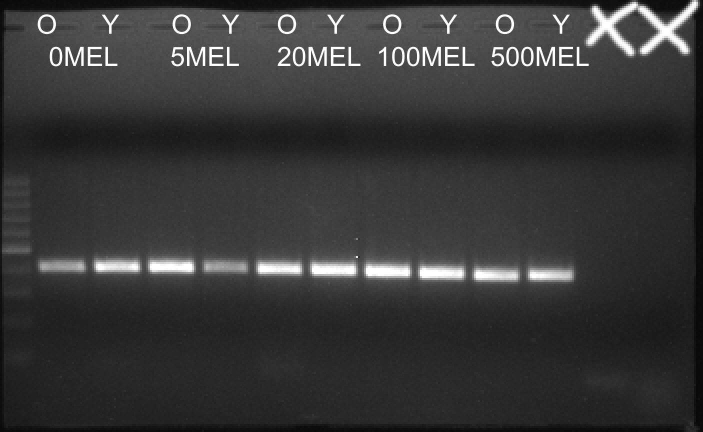

SrMCT

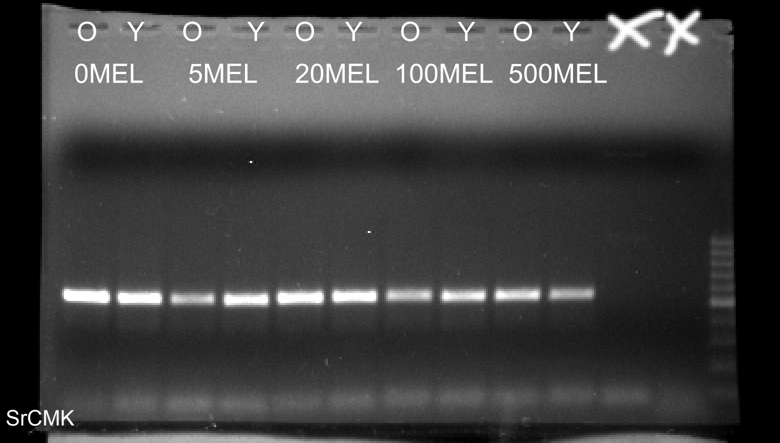
O Y O Y O Y O Y O Y
0MEL 5MEL 20MEL 100MEL 500MEL
SrCMK

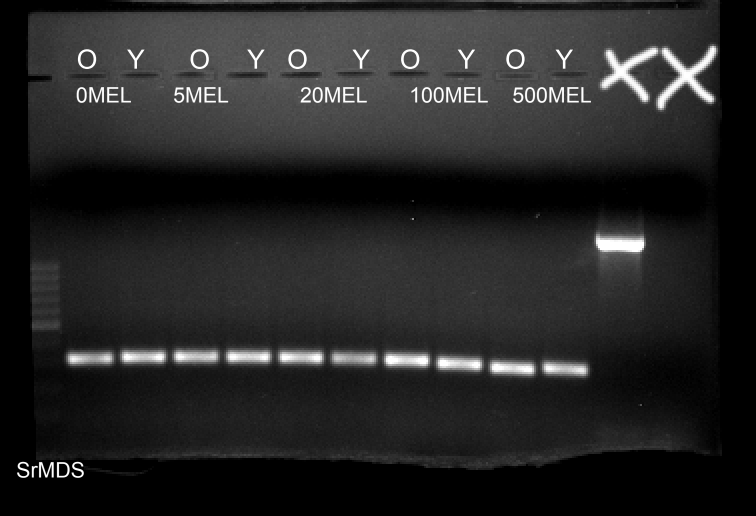
O Y O Y O Y O Y O Y
0MEL 5MEL 20MEL 100MEL 500MEL
SrMDS

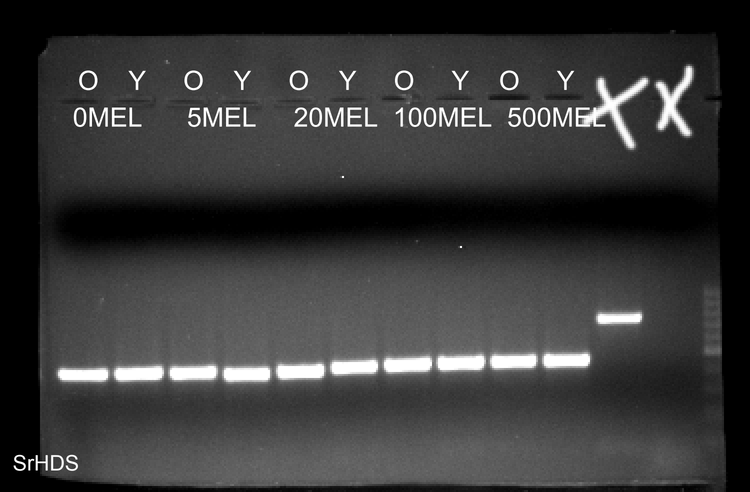
O Y O Y O Y O Y O Y
0MEL 5MEL 20MEL 100MEL 500MEL
SrHDS

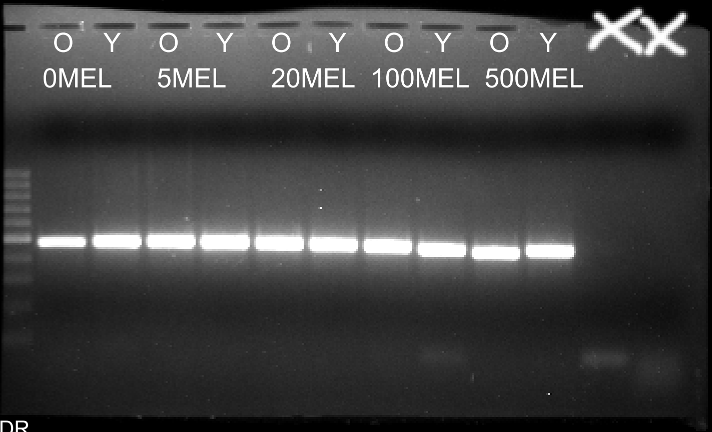

SrHDR

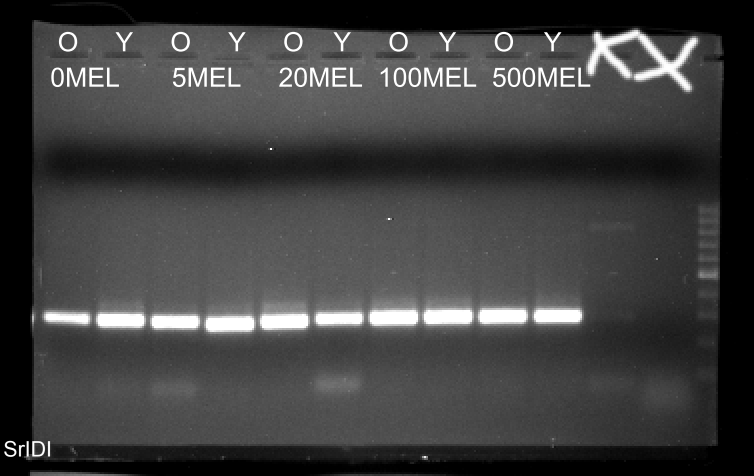
O Y O Y O Y O Y O Y
0MEL
5MEL
20MEL
100MEL
500MEL
SrIDI

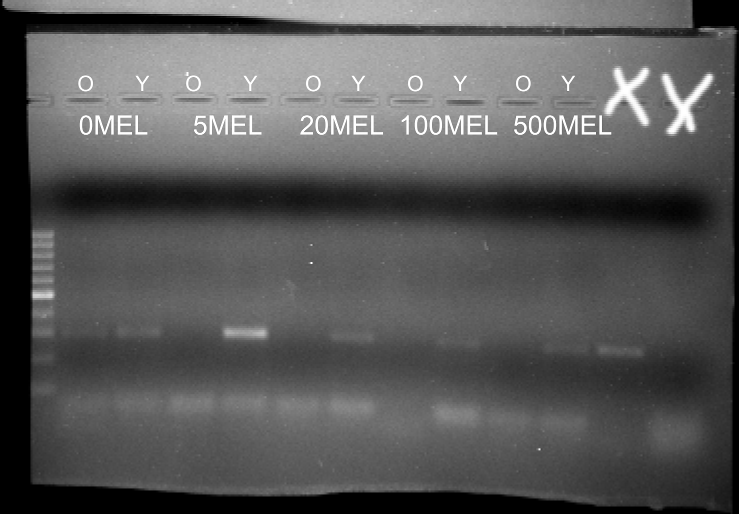

SrGGDPS

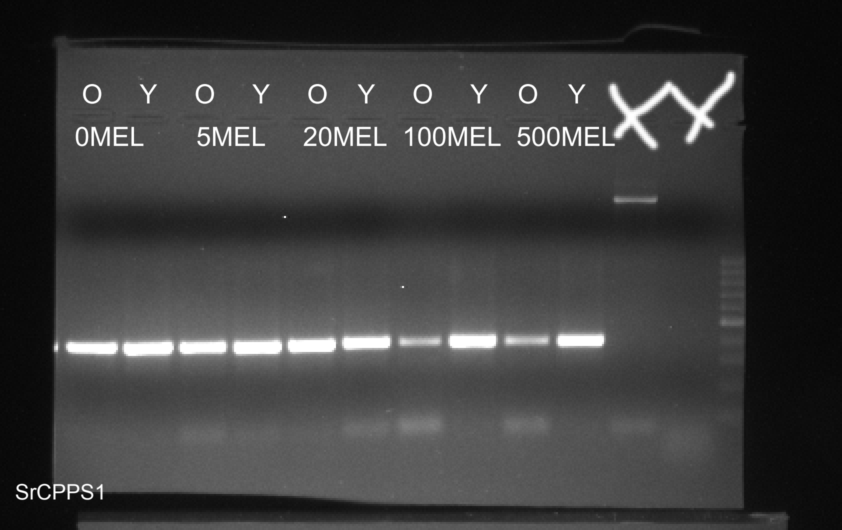
O Y O Y O Y O Y O Y
0MEL
5MEL
20MEL
100MEL
500MEL
SrCPPS1

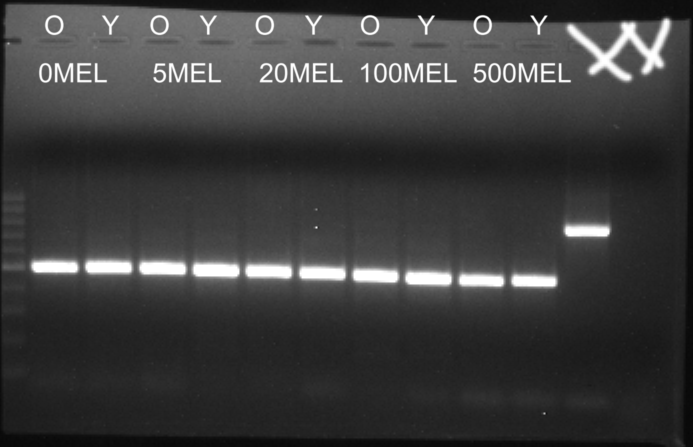

SrKS1

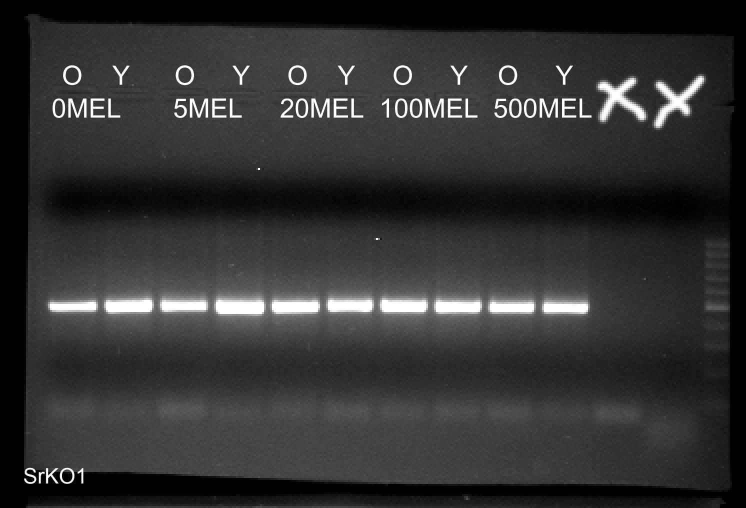
O Y O Y O Y O Y O Y
0MEL
5MEL
20MEL
100MEL
500MEL
SrKO1

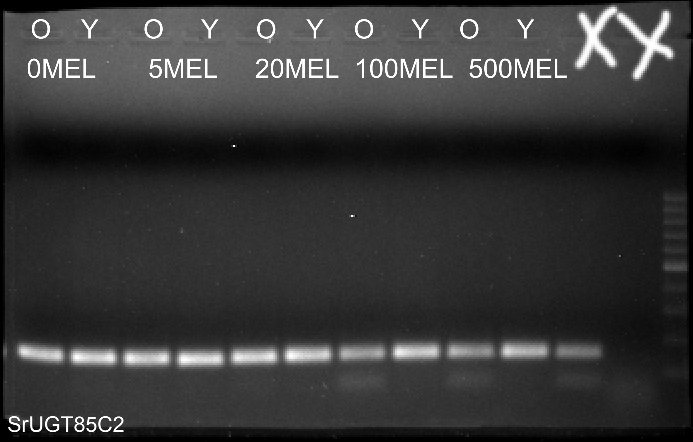
O Y O Y O Y O Y O Y
0MEL 5MEL 20MEL 100MEL 500MEL
XY
SrUGT85C2

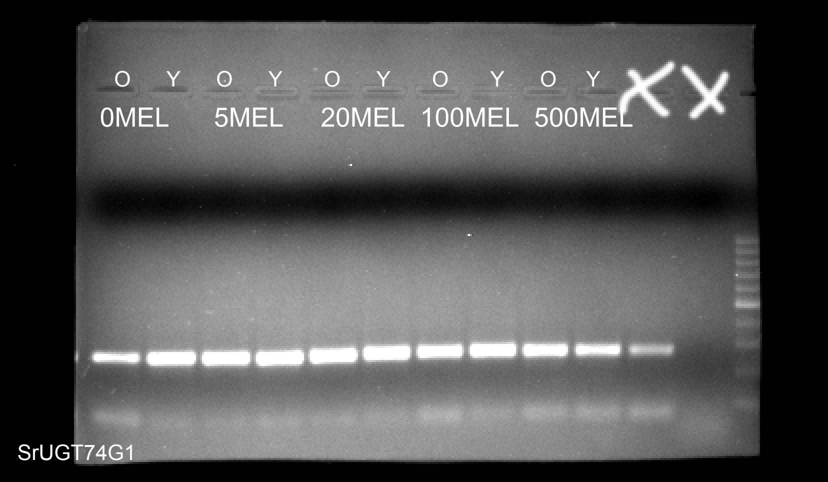
O Y O Y O Y O Y O Y
0MEL
5MEL
20MEL
100MEL
500MEL
SrUGT74G1

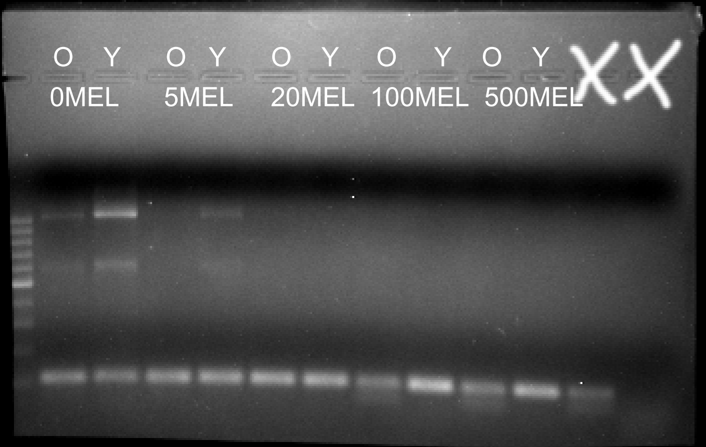

SrUGT76G1
