# Supplementary material for: Melatonin promotes seed germination under salinity and enhances the biosynthesis of steviol glycosides in Stevia rebaudiana Bertoni leaves
Source: PLoS One. 2020 Mar 27;15(3):e0230755. doi: 10.1371/journal.pone.0230755 (PMC7100979; doi:10.1371/journal.pone.0230755)
Supplement: S4 Raw Images — (PDF) [file pone.0230755.s008.pdf]

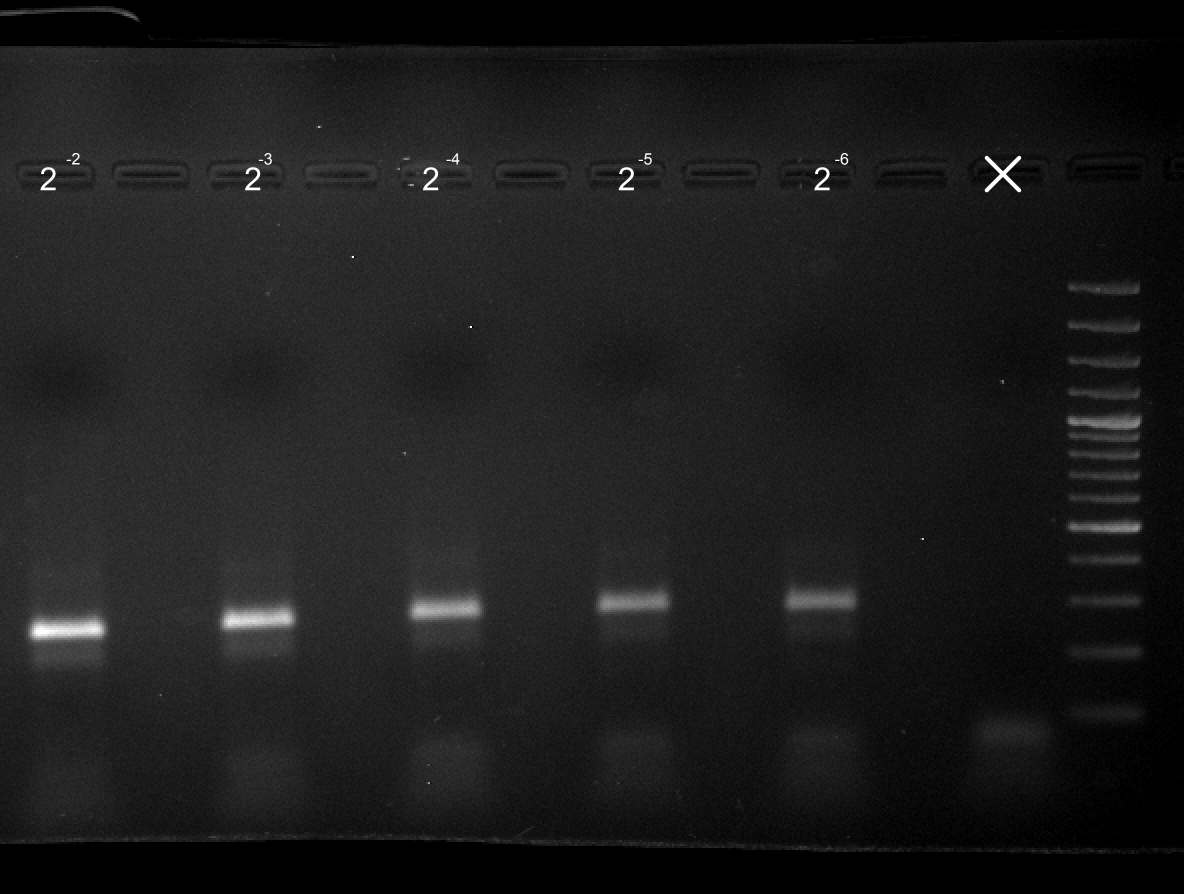

2^{-2}
2^{-3}
2^{-4}
2^{-5}
2^{-6}
X
